# Efficient and rapid generation of large genomic variants in rats and mice using CRISMERE

**DOI:** 10.1038/srep43331

**Published:** 2017-03-07

**Authors:** Marie-Christine Birling, Laurence Schaeffer, Philippe André, Loic Lindner, Damien Maréchal, Abdel Ayadi, Tania Sorg, Guillaume Pavlovic, Yann Hérault

**Affiliations:** 1 PHENOMIN, Institut Clinique de la Souris (ICS), CNRS, INSERM, University of Strasbourg, 1 rue Laurent Fries, F-67404 Illkirch-Graffenstaden, France; 2 Institut de Génétique et de Biologie Moléculaire et Cellulaire, Illkirch, France; 3 Centre National de la Recherche Scientifique, UMR7104, Illkirch, France; 4 Institut National de la Santé et de la Recherche Médicale, U964, Illkirch, France; 5 Université de Strasbourg, Illkirch, France

## Abstract

Modelling Down syndrome (DS) in mouse has been crucial for the understanding of the disease and the evaluation of therapeutic targets. Nevertheless, the modelling so far has been limited to the mouse and, even in this model, generating duplication of genomic regions has been labour intensive and time consuming. We developed the CRISpr MEdiated REarrangement (CRISMERE) strategy, which takes advantage of the CRISPR/Cas9 system, to generate most of the desired rearrangements from a single experiment at much lower expenses and in less than 9 months. Deletions, duplications, and inversions of genomic regions as large as 24.4 Mb in rat and mouse founders were observed and germ line transmission was confirmed for fragment as large as 3.6 Mb. Interestingly we have been able to recover duplicated regions from founders in which we only detected deletions. CRISMERE is even more powerful than anticipated it allows the scientific community to manipulate the rodent and probably other genomes in a fast and efficient manner which was not possible before.

Mouse models have been key elements to better understand the physiopathology of Down syndrome (DS)^1^,^2^,^3^,^4^,^5^,^6^. Nevertheless new models in rat may offer opportunities for exploring behaviour, cognition, memory and social interaction. In addition, being separated from mouse for more than 11 million years the rat represent an alternate model of choice. Mouse, rat and human share many homologies at the genomic^7^,^8^,^9^,^10^ and physiological levels so that conclusions drawn in two rodent species would be stronger for translational approach in human. More than 200 protein coding genes have been identified on human chromosome 21 (HSA21), for which 188 orthologs have been found on rat chromosomes 11 and 20 (www.ensembl.org/biomart; see Fig. 1). For example, a large fragment ranging from *Lipi* to *Zfp295* encompassing 24.4 Mb and 113 protein-coding genes is located on rat chromosome 11 (RNO11; RNO for *Rattus norvegicus*). It is similar to the region located on the mouse chromosome 16. The most telomeric part of HSA21 long arm is located on a unique segment on the RNO20, with 74 genes found between *Umodl1* and *Prmt2*. By contrast, the mouse orthologs for these genes are present in segments of two chromosomes in the mouse^11^. On this basis, we focused on developing rat models to decode the cellular and molecular mechanisms linked to the neuropathological, cognitive, physiological, and morphological alterations found in human DS patients.

So far the lack of embryonic stem cell was a limiting step for generating DS rat model. Indeed modelling segmental duplication has been achieved so far by using the Cre-lox technology^12^,^13^,^14^,^15^ in combination with ES cells or alternative strategies^16^,^17^,^18^ only in mouse. Briefly, this strategy requires the generation of two mouse lines with loxP site inserted in the same orientation at the border of the region of interest, followed by 3-step breeding to introduce a Cre driver expressed in the germ line^15^,^14^. For inversions, new experimental design and mouse lines had to be generated^19^ with inverted loxP sites. The overall strategy necessitates at least 3 years to generate a model but demonstrated the power of a mapping panel of duplication to better understand the contribution of HSA21 regions to DS phenotypes^3^. Recently, CRISPR/Cas9 was used to manipulate large genomic region in the mouse genome. The Wu lab^20^ demonstrated that DNA elements of up to 29 kb can be manipulated directly in mice using the CRISPR/Cas9 technology. Zheng and Li’s lab showed that deletion of up to 95 kb can be generated in mice^21^,^22^. Similarly, gene clusters of up to 800 kb can be deleted, duplicated, and inversed in HEK293T cells^23^. CRISPR/Cas9 was used for the generation of up to 1 Mb structural deletion and inversion around the Tyrosinase locus^24^ in mouse zygotes but duplications appeared less frequently and did not pass the germ line.

In this paper, we investigated how mouse or rat segmental duplications, and other large scale rearrangements, can be easily generated using the CRISPR/cas9 technology in zygotes either for a single candidate gene or for larger to very large genomic regions. These models and their analyses will allow us to better understand the complexity of the genetic interactions that are involved in DS cognitive phenotypes and could challenge the hypothesis regarding the multigenic nature of DS in a new species.

## Results

### Successful segmental inversion, deletion and duplications of two rat genes by CRISMERE

We defined the CRISMERE approach by using two pairs of sgRNAs, one pair located upstream and the other pair downstream of the region of interest (called here break site), for all the experiments described in this paper. All sgRNAs microinjected were selected and validated *in vitro* (see Methods) on a PCR fragment containing the target sequence, but we (like others) observed that the *in vivo* efficiency can vary from one sgRNA to another. For our first attempt, the pairs of sgRNA were used in combination with the D10A nickase Cas9 mutant but no chromosomal rearrangement was observed. When the same guides were used with wild type (wt) *Streptococcus pyogenes* Cas9 the occurrence of rearrangement increased substantially. We decided to keep this configuration for our subsequent experiments. The two sgRNAs of a pair recognized close sequences (distance between two guides was always lower than 150 bps) to increase the probability to obtain a double strand break (DSB) (see Supplementary Data).

**Figure 1:**
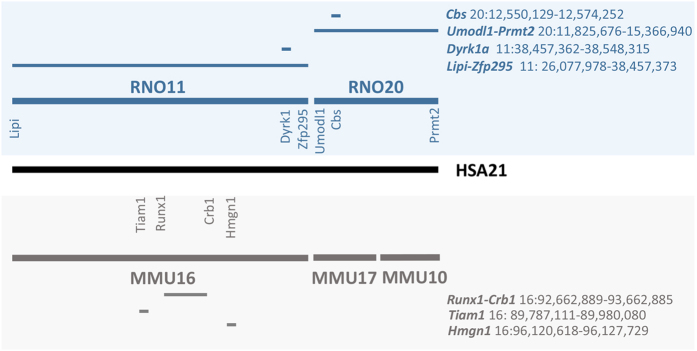
Genetic regions triplicated in DS mouse models. The relative position of the mouse homologous regions on MMU16, −17, −10 and rat homologous region on RN011 and −20 to HSA21 are shown with the genes located at the borders of the genetic interval. In blue and grey, the position of the DS models (rat or mouse respectively) attempted in this paper.

**Figure 2:**
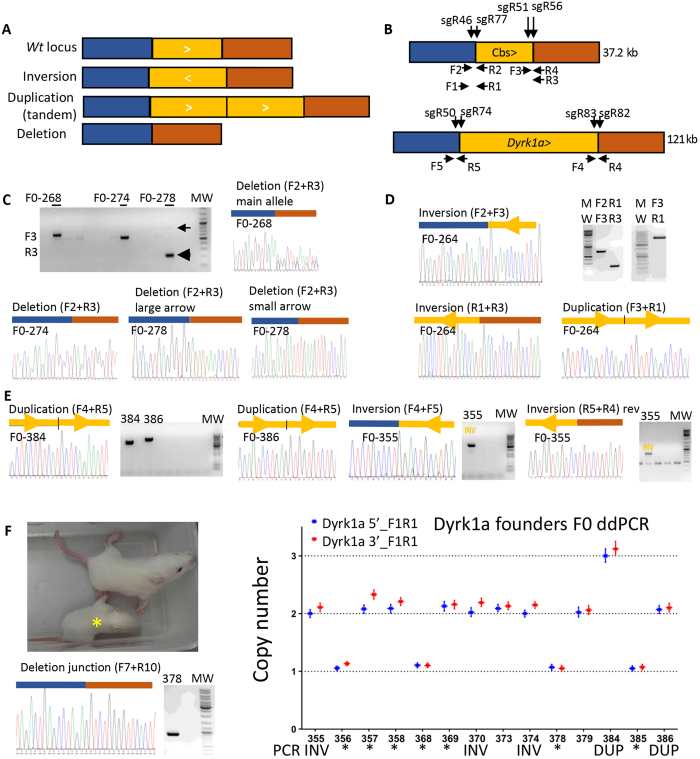
Targeted deletion, duplication and inversion for two rat genes. (**A**) Variety of alleles detected in founders F0 and F1 when two pairs of sgRNAs for two sites flanking a region of interest are used. In blue the genomic region 5′ of the region of interest, in yellow the region of interest and in orange the genomic region 3′ of the region of interest. (**B**) Diagram for rat *Cbs* and *Dyrk1a* genes. Shown are the position of the primers used for genotyping, and position of the sgRNAs (**C**,**D**) concern *Cbs*. (**C**) Shown are deletion junction chromatograms of 3 different founders. Only one deleted allele was obtained in F0-274 (one band detected by PCR) whereas 2 deleted alleles were detected for founders F0-268 and -278 (2 bands observed by PCR and confirmed by a close analyze of the chromatograms). For founder F0-278, a bold arrow faint arrow shows the two distinct deletion alleles (see also Supplementary data S1). Both alleles passed germ line. (**D**) Shown are duplication and inversion junctions for *Cbs* amplified by PCR from ear clipping rat founder F0-264 with specific primers pairs. An example of sequence chromatograms of the inversion and duplication junctions is shown. The same inversion and duplication events were found independently on F1 rats (**E**,**F**) concern Dyrk1a (**E**). The duplication junctions amplified by PCR from ear clipping rat founders F0-384 and F0-386 with specific primers pairs are shown. Inversion junctions of F0-355 founder is shown as an example (see also Supplementary data S2) (**F**) Detection of rats with one copy of *Dyrk1a*. Appearance of a small founder F0 (yellow arrow) compared to its wt littermate. Monosomic rats for *Dyrk1a* were first detected by droplet digital PCR. Two ddPCRs (in the first and last exon) were performed in *Dyrk1a* gene and the loss of one complete copy was confirmed for F0-356, -368, -378 and F0-385. The junction for F0-378 is detected only with primers located farther from the sgRNAs and shows a more important deletion than expected (see Supplementary Fig. S1).

First we selected the locus of the *cystathionine beta synthase* gene, *Cbs*, located in a region contributing to DS phenotypes in mouse models^25^,^26^. In human, *CBS* recessive mutation deficiency leads to homocystinuria (OMIM 236200). *Cbs* homozygous knock-out in mouse are severely growth retarded and die within 5 weeks of birth while heterozygotes survive^27^. In rat, *Cbs* (ENSRNOG00000029528) is located on RNO20 in the syntenic HSA21 interval (Fig. 1). The generation of *Cbs* structural variant models was tested by CRISMERE. We designed two pairs of sgRNAs targeting both extremities of the 37.2 kb region containing the *Cbs* gene (for sequence details see Supplementary Table 1 and Supplementary Data S1). Injection conditions and genotyping results are synthetized in Table 1. Given that DNA duplications, deletions, and inversions have two junctions, we designed two pairs of specific PCR primers near the cleavage sites of the Cas9 to identify upstream and downstream DSB (Fig. 2B). We used different combinations to identify inversion, deletion, and duplication junctions to make sure we would detect any event (Fig. 2A and B). Twenty-four F0 founder rats were born and screened by PCR at 4 weeks for the whole variety of potential alleles. We detected 12 F0 founders (50%; 12 out of 24) with a deletion of the whole *Cbs* gene (for details see Fig. 2C and Supplementary Data S1). Interestingly, five F0 founders showed two deletion on two alleles, of distinct sizes and intensity, which were confirmed by Sanger sequencing. For instance, rat founder F0-278 had two different deleted alleles (close and large arrows on Fig. 1B; see also Supplementary Data S1) that went germline. The deletion annotated by the faint arrow occurred frequently (12/25 F1 pups) while the other deletion was only detected in one F1 pups (1/25). Inversion events were less frequent (7 F0 rats; 29%) but still abundant. Only one F0 founder had detectable tandem duplication (4.2%) (F0-264; Fig. 2D). This duplication was transmitted to the F1 (3/14 pups). The inverted allele observed in the same founder (Fig. 2D and Supplementary Data S1) was also transmitted to the germ line at lower frequency (3/21 pups).

We next investigated a second locus for the *Dual-specificity tyrosine phosphorylation-regulated kinase 1a (Dyrk1a*). In the last decade, *DYRK1a* has become one of the top candidate gene in DS for therapeutic intervention^28^,^29^. It is mutated in the Mental Retardation Autosomal Dominant 7 (MRD7, OMIM 614104)^30^,^31^,^32^. This gene is located within the DS-critical region on HSA21 (ENSG00000157540; see Fig. 1). Transgenic *Dyrk1a* mouse models exist and have already provided key information on the role of this gene^33^,^34^. The homozygous *Dyrk1a*-deleted (*Dyrk1a*^−/−^) mice present a general embryonic growth delay and die during mid gestation. Heterozygous *Dyrk1a*^+/−^ mice display reduced postnatal survival, postnatal growth retardation, microcephaly, behavioural and motor deficits, and altered neocortical pyramidal cell morphology^35^,^36^,^37^,^38^. As no rat models exist, the availability of *Dyrk1a* trisomic or monosomic rat models would be valuable tools for understanding this gene’s function in DS and MRD7. The rat *Dyrk1a* (ENSRNOG00000001662) is located on RNO11 (Fig. 1). We designed two pairs of sgRNAs in order to generate structural variants of rat *Dyrk1a* gene (see Fig. 2B and Supplementary Table 1). The size of the whole targeted region was 121.7 kb. Injection conditions are described in Table 1. We observed 7.4% (2 out of 28) of the pups showing the 121.7 kb duplication (Fig. 2E) and three of them (10.7%; 3 out of 28 pups) showed a clear inversion (Fig. 2E). We were not able to detect the deletion of the region by standard PCR using six combinations of primers (F1 + R3 or R4; F3 + R3 or R4; F5 + R3 or R4, see Supplementary Table 1). This was most surprising as we clearly observed that seven of the potential founder pups were significantly smaller than wild-type rat at early post-natal time (2–3 weeks; Fig. 2F) reminding us the phenotype observed in *Dyrk1a*^+/−^ mice^35^,^36^,^37^,^38^. We suspected a failure in the amplification of the junction PCR detecting the deletion. We, therefore, decided to use droplet digital PCR (ddPCR) to quantify *Dyrk1a* allele copy number. From this, we were able to confirm one of the duplication events (F0-384) but not the other (F0-386) (Fig. 2F). Germline transmission was confirmed for founder F0-384 using both ddPCR and junction sequencing. In founder F0-386, allele counting demonstrated that *Dyrk1a* remained at two copies, suggesting that one allele of *Dyrk1a* is deleted whereas the second allele is duplicated in most of the F0 founder cells. Most striking was the confirmation of the presence of a single copy of *Dyrk1a* in four out of the seven small rats (Fig. 2F). We were not able to detect any modification in the three remaining small rats by ddPCR or junction sequencing. Again, this might be due to mosaicism rendering copy counting difficult or other undetectable chromosomic rearrangement. As expected, inversions were not detected by ddPCR, the number of copy remains stable when an inversion occurs. Founders were weighted at ten-eleven weeks. We observed a clear reduction in weight (average of 368 grams) for the 4 founders with one *Dyrk1a* copy (ddPCR detection) compared to the other founders (average of 464 grams) when two *Dyrk1a* copies detected by ddPCR (Supplementary Fig. 1A). Despite its low weight, the male F0-378 (1 copy detected by ddPCR) fertilized a female before undergoing euthanasia (very bad general state) at 10 weeks of age. We collected tissue samples, purified genomic DNA and performed long range PCR in order to assess the precise junction of the deletion. We selected new primers farther to the expected break sites and were able to amplify and sequence the junction (Supplementary Fig. 1B and D and Supplementary Data). The size of the region deleted was indeed larger than expected and explained why we were not able to detect it with the standard primers used initially. Three F1 pups (out of 8) transmitted the deletion as confirmed by ddPCR and junction PCR (Supplementary Fig. 1C and D). As expected, these rats are 40% smaller than their wt littermates (Supplementary Fig. 1E).

### Generation of large regions structural variant ranging from 3.6 Mb to 24.4 Mb in rat

**Figure 3:**
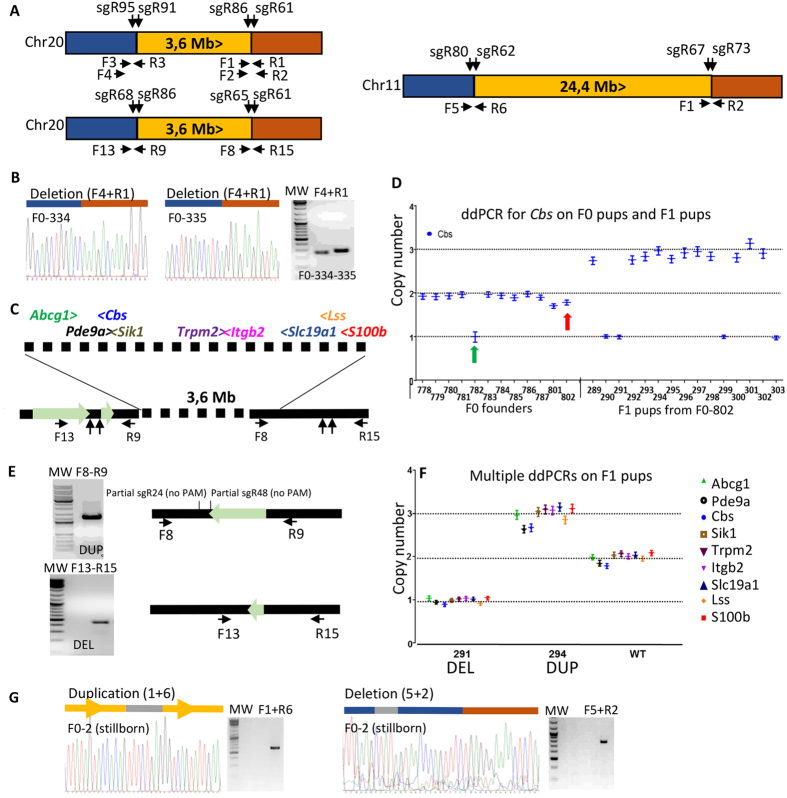
Targeted deletion and duplication for two rat genomic regions. (**A**) Diagrams show both regions of interest (in orange, surrounded by 5′ (blue) and 3′ (brown) regions). Two pairs of sgRNAs flank both regions of interest. Shown are the position of the primers used for genotyping and position of the sgRNAs. (**B**) Targeted deletion of *Umodl2-Prmt2* region (3.6 Mb) in founders. Shown the deletion junction chromatograms obtained after PCR amplification with specific primers pairs (F4 + R1) in rat founders F0-334 and F0-335. (**C**) Scheme of the 3.6 Mb genomic region with zoom at both extremities. Positions of the sgRNAs and PCR primers are illustrated. ddPCRs were performed on 9 genes (*Abcg1*, green; *Pde9a*, black; *Cbs*, blue; *Sik1*, brown; *Trpm2*, purple; *Itgb2*, pink; *Slc19a1*, dark blue; *Lss*, orange and *S100b*, red) distributed over the 3.6 Mb region. The region highlighted with a large light green arrow is found at the junction of the duplication. Oligonucleotides used (F and R) are shown (**D**) Droplet digital PCR results. Left panel of the graph shows some F0 results: a single copy of *Cbs* was detected in F0-782 whereas 1.8 copies of *Cbs* were detected in F0-802. On the right panal are F1 results: 1 or 3 copies of *Cbs* were clearly detected on the F0-802 first litter showing that both deletion and duplication went germ line. (**E**) Confirmation of the tandem duplication and deletion by junction sequencing. A 590 bp inversed fragment corresponding to the region 5′ of the first pair of sgRNA is found at the duplication junction. A small (70 bp) inversed sequence is found at the deletion junction (see Supplementary data S3D and E for details) (**F**) ddPCR results with 9 primer pairs on 2 F1 pups (291-DEL and 294-DUP) confirmed the deletion and duplication of the whole genomic region; a WT rat was taken as control (2 copies) (**G**) Targeted deletion and duplication of >24 Mb region in a stillborn rat F0. Shown are duplication and deletion junctions amplified by PCR on a stillborn rat F0 (Supplementary nts in gray). The gel pictures of both duplication and deletion junctions are presented.

We wanted to assess if larger chromosomic rearrangements are also possible. We targeted the rat 3.6 Mb region spanning from *Umodl1* (ENSRNOG00000001157) to *Prmt2* (ENSRNOG00000001297) located on RNO20 and encompassing the most telomeric part of the HSA21. This region is the second larger syntenic region to HSA21 (see Fig. 1). Two sgRNA pairs targeting the 5′ region of *Umodl1* at the most telomeric part (sgRNA95 and sgRNA91) and the 3′ region of *Prmt2* (sgRNA86 and sgRNA61) were synthesized (Fig. 3A; Supplementary Table 1 and Supplementary Data S3A). Injection conditions are described in Table 1. We observed 5% (2 out of 40 pups) pups showing a 3.6 Mb deletion (Fig. 2B). The experiment showed that manipulation of large genomic sequence is feasible. No germ line transmission was achieved. Another set of experiments with two different sgRNA pairs was performed (Fig. 3C; Supplementary Table 1 and Supplementary Data S3C). Rat pups born after microinjection were screened by ddPCR (Fig. 3D) and junction PCR. One copy of *Cbs* (gene located in the region) was clearly detected in F0-782 whereas 1.7 and 1.8 copies were counted in F0-801 and F0-802 (Fig. 3D, left panel). No PCR products were obtained with junction PCRs. These 3 founders were bred in order to analyse their offspring. Germ line transmission of both the duplication and deletion was obtained from F0-802 (Fig. 3D, right panel). Both junctions were determined by junction PCR and sequencing (Fig. 3F and E and Supplementary Data S3). Nine different ddPCRs were performed on different gene present on the 3.6 Mb fragment, all confirmed that the whole region was indeed duplicated or deleted (Fig. 3F).

A larger 24.4 Mb chromosomal rearrangement was then attempted to recapitulate the human largest syntenic region to HSA21 (spanning from *Lipi* to *Zfp295*, see Fig. 1). We designed two pairs of sgRNAs for upstream and downstream sites flanking the rat 24.4 Mb DNA region located on RNO11 (Fig. 3A; Table 1; Supplementary Data S4 and Supplementary Table 1). Different combinations of primers were used to identify inversion, deletion, and duplication junctions (Fig. 3A and Supplementary data S4A). The monosomy of this 24.4 Mb region induces lethality in mice but the trisomy is viable both in mice^39^ and human (Down syndrome patients). We took care to genotype the stillborn rat pups. On the unique stillborn founder F0, we were able to detect both the deletion and tandem duplication of the 24.4 Mb region by junction PCR and Sanger sequencing (Fig. 3G and Supplementary Data S4). In addition, small deletions at both break points were also detected in the same stillborn rat (Supplementary Data S4B). A deletion of 51 bps was found near *Lipi* between the two DSBs induced by the Cas9 3 bps upstream of the PAM sites. A deletion of 35 bps was found between the two DSBs accompanied with a short insertion of 10 nts in near *Zfp295*. This shows the high efficiency of the sgRNAs at both extremities. Eight out of the 9 founders had indels at a least one or the other break points. Subsequently, we performed injections in order to obtain the duplication but have not been able to obtain a surviving founder F0 with the duplication only. These experiments highlight the limit of our approach, namely the inherent viability problems of the variant generated in the rodent. We are currently assessing if duplication can occur in the germ line cells of founder F0s by breeding a few founders in which we detected DSBs at both 5′ and 3′ break points.

We show with these four examples that any kind of chromosomal rearrangement of any size can be fairly easily achieved in rat using CRISPR/Cas9. The only limitation we find is the lethality of the variant generated. Indeed, our evidence suggests that the larger the genomic fragment, the higher the risk to cause lethality.

### Generation of structural variants of various size in mouse

We have demonstrated that the CRISMERE strategy in rat can be easily used to manipulate large genomic regions. Thus, we decided to test the strategy in mouse to generate a panel of DS models.

**Figure 4:**
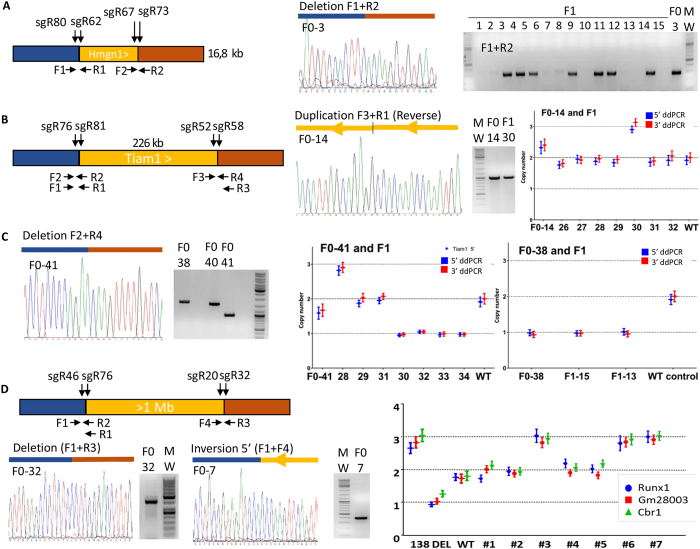
Mouse models obtained by CRISMERE. Diagrams are as in Fig. 3. Shown are junctions amplified by PCR from mouse tail with specific primers. Examples of sequence chromatograms of the different junctions are shown. (**A**) *Generation of monosomic Hmgn1 mouse line*. The chromatogram of the deletion junction observed in F0-3 is shown (details in supplementary data S6). Germ line transmission was confirmed in 7 out of 15 offspring. (**B**,**C**) *Deletion and duplication of mouse Tiam1 (226 kb*). (**B**) Shown the chromatogram of the duplication junction observed in F0-14, the gel picture of the PCR products from F0-14 and a F1-30. Graph showing ddPCR results on both F0-14, 7 F1 offspring and a WT animal. F0-14 mosaicism is shown as 2.3 *Tiam1* copies are detected with 2 distinct ddPCRs (5′ and 3′ of Tiam1, red and blue dots). Germ line transmission was confirmed by ddPCRs (3 copies detected in F1-30). (**C**) Shown the chromatogram of the deletion junction (primers F2 + R4) of F0-41 and gel picture with the PCR for F0-38, F0-40 and F0-41. Germ line transmission for the deletion was confirmed by ddPCR (F1-30 to F1-34; one copy detected) whereas the founder (F0-41) had a mosaic profile (1.7 copies of *Tiam1*). Note also a duplicated allele (3 copies of *Tiam1*) is also detected in F0-41 offspring (F1-28). A single copy of *Tiam1* was detected in F0-38 by ddPCR (next graph) suggesting absence of mosaicism. The same ddPCR profile was observed for 2 of the offspring (F1-15 and -13). (**D**) *Targeted deletion and inversion of the mouse genomic region ranging from Runx1 to Cbr1 (1 Mb*). Shown the chromatogram of the deletion junction amplified by PCR from F0-32 tail (F1 + R3) with accompanying gel picture. Inversion of the whole region is confirmed by PCR amplification and Sanger sequencing of the 5′ junction on F0-7 (F1 + F4). Droplet digital PCRs on 3 genes distributed over the 1 Mb region (*Runx1*, blue; *Gm28003*, red and *Cbr1*, green) detected 3 copies in F0-138 as well as some of its offspring (F1-3, -6 and -7). A WT control (2 copies) and a deletion (1 copy) are included.

First, we attempted the deletion and duplication of the *high mobility group nucleosomal binding domain 1 (Hmgn1;* ENSMUSG00000040681), a 16.8 kb locus (Fig. 1). In human, overexpression of *HMGN1* (ENSG00000205581), a nucleosome remodelling protein encoded on chromosome 21q22^40^, suppresses histone 3 lysine 27 trimethylation (H3K27me3) and promotes both B cell proliferation *in vitro* and B cell acute lymphoblastic leukemia *in vivo*^41^. Two pairs of sgRNAs surrounding the murine *Hmgn1* sequence were synthesized (see Fig. 4A, Supplementary Data S5 and Supplementary Table 1). Injection conditions are described in Table 1. We were able to detect the deleted allele in 4 out of 8 founders (50%). Two founders were bred with wild type animals and both gave germline transmission (founder F0-3: 13 F1 out of 49 (26%); founder F0-8: 4 F1 out of 20 (20%) (Fig. 4A). These mice are currently breeding to establish phenotyping cohorts.

Next we focused on the *T-cell lymphoma invasion and metastasis 1* gene (*TIAM1*; ENSG00000156299). Indeed TIAM1 is located on HSA21 and has been shown to be a critical regulator of different aspects of *Ras*-induced tumour formation^42^. We aimed to generate the deletion and duplication of the whole murine *Tiam1* gene (ENSMUSG00000002489) a region spanning 226 kb. We were mainly interested in the trisomic version of this gene as the monosomic model (i.e. heterozygous knock-out mice) has already been studied^42^. We thus decided to duplicate the *Tiam1* gene using the double sgRNA pair strategy. Microinjection conditions can be found in Table 1. Forty one mouse pups were born and PCR primer pairs were designed to detect all the combination of alleles (see Fig. 4B). We observed 19.5% (8 out of 41) of the pups showing a 226 kb deletion and one of them showed a clear duplication (Fig. 4B) (2.4%; 1 out of 41 pups) (Table 1). No inversions were detected by junction PCR. Three founders with a deleted allele were bred with wild type C57BL/6 N mice and germ line transmission was obtained for each of them (Fig. 4C). Junction sequencing of F1 mice confirmed the deletion observed in founders. Seventeen F1 pups were born from founder F0-38, seven (41%) had the deletion. The *Tiam1* deletion was also confirmed by droplet digital PCR (Fig. 4C) with two different pairs of primers specific from both extremities of the *Tiam1* gene. Interestingly, ddPCR confirmed that founder F0-38 was carrying mostly heterozygous cells as only one copy was detected. This mutant allele also demonstrated a high Mendelian transmission rate observed (41%, close to the 50% rate expected). Thirty-four mice were born after breeding the founder F0-41 and only eight F1 (23%) had the deleted allele. ddPCR copy counting (1.7 *Tiam1* copy number, Fig. 4C) confirmed the mosaic nature of this animal. Interestingly, we were able to detect 3 *Tiam1* copy number in one F1 mouse (out of 7 F1 pups analysed by ddPCR). Thus, a duplication event can be transmitted to the germ line that is not necessarily detected in a founder F0 (see Fig. 4C, mouse 28). Considering that we were not able to detect this duplication by standard junction genotyping, this highlights the complementarity of both genotyping techniques. Finally, the F0-14 founder for which we detected a duplication by Sanger sequencing also showed a mosaic pattern by ddPCR (2.3 copies detected), we obtained only one single F1 pups with the same duplication (out of 33 genotyped) showing that the founder was highly mosaic. We demonstrate here that ddPCR allows us to detect deletions and duplications in F1 or later generations and eliminates the possibility of PCR failures (as the PCR is not performed at the junctions but inside the copy number variant). However ddPCR fails to detect inversions and rare/mosaic rearrangement and some mosaic founders (del/dup in G1, see discussion). Regardless of the means of detection, we have demonstrated that DNA region deletion or duplication by CRISPR with two pairs of sgRNAs can be transmitted through germline in mice.

We next focused on the 1 Mb long mouse genomic region comprising three genes (*Runx1, Setd4* and *Cbr1*) (see Fig. 1). Two lincRNAs are also located in this interval (*1810053B23Rik* and *1700029J03Rik*). *Runx1* homozygote knockouts die by embryonic day 12.5 from a complete lack of definitive hematopoiesis and lethal central nervous system (CNS) hemorrhage^43^ whereas heterozygotes have reduced erythroid and myeloid progenitor numbers. Two pairs of sgRNAs surrounding this region were chosen (Fig. 4D and Supplementary Data S6). Microinjection conditions are described in Table 1. We were able to genotype one founder with an inversion of the 1.1 Mb region (3% of genotyped pups) as confirmed by Sanger sequencing of the 5′ junction (Fig. 4D). Another F0 founder (F0-32) showed a confirmed deletion (3%). This F0 pup died after 3 weeks. We performed another round of microinjection with the same sgRNAs and obtained founders with one copy of the region and copy numbers ranging from 1 to 2.6 (data not shown). We obtained germ line transmission for the deletion (1 copy by ddPCR; 3 genes spread across the 1.1 Mb region- Runx1, Gm28003 and Cbr1) and the duplication (3 copies by ddPCR, Fig. 4D). Altogether, these data definitively demonstrated for the first time that segmental duplication, deletion, or inversion of any size can be efficiently induced in rodents by CRISMERE with two pairs of sgRNAs (see Table 1 for summary).

## Discussion

In this study we have shown that any kind of chromosomal rearrangements can be generated in both rat and mouse using CRISMERE. The rearrangements ranged in size from a few kilobases to more than 24 megabases and were achieved with relative ease *in vivo* for rat and mice. Our method for generating DNA fragment inversions, deletions and duplications is simple and efficient. The efficiency of these rearrangements mediated by CRISPR/Cas9 is a significant improvement over methods through recombinases and nucleases^15^,^20^,^44^,^45^,^46^ paving the way for efficient DNA fragment inversion, deletion, and duplication in any species *in vivo*. Indeed, editing large DNA fragments of several hundred kb can be used to model DNA segmental duplications, which are common in mammalian genomes^47^. Previously, the generation of such a model was possible only in a very narrow range of species, very time consuming, expensive, and limited in the variety of rearrangements one sought to mimic among the many observed in human disease. Now with the CRISMERE strategy described above such chromosomal modifications should be straightforward.

**Figure 5:**
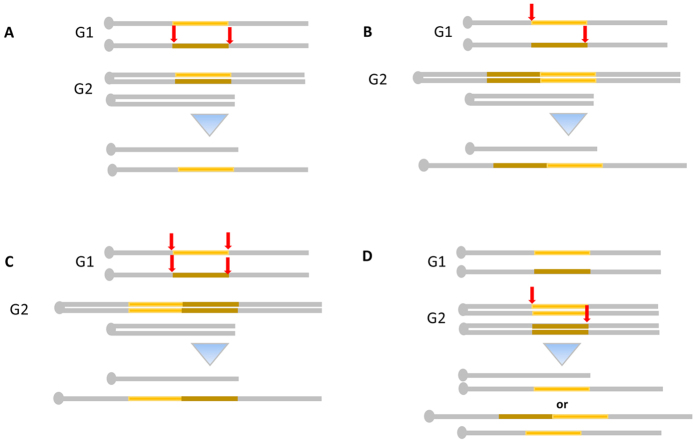
CRISpr MEdiated REarrangement mecanisms (CRISMERE) sgRNA pairs are illustrated by red arrows. Chromosomes are represented by a thick gray line, the centromeres by a gray dot and the region of interest in yellow. (**A**–**C**) Standard chromosomic recombination when Cas9 edits the genome in G1. After mitosis, two alleles distinct from the initial WT allele will be obtained (**A**) Intra-chromosomal recombination between two DSBs on a single chromosome (**B**) Trans-allelic recombination between two DSBs each on one of the two chromosome. (**C**) Trans-allelic recombination between (three or) four DSBs on the two chromosomes ending with head to head, tail to tail duplication (**D**) Schematic of the event that should take place in the eggs were Cas9 edits the genome in *Cis* configuration in G2 leading to monosomic and trisomic daughter cells after mitosis. Trans-allelic recombination between two DSBs on the two chromatids in G2 (similar to targeted asymmetric sister chromatid event of recombination TASCER).

According to our results there are two possibilities to obtain genomic DNA region rearrangements for two concomitant DSBs (here generated by pairs of sgRNAs): intra-chromosomal recombination between two DSBs on a single chromosome (Fig. 5A) and trans-allelic recombination between two DSBs each on one of the two chromosomes (Fig. 5B). These events must happen in mitotic phase G1 before the chromosome replication. The range of events that can be obtained with CRISMERE is even broader than what was first expected. Indeed, three or four DSBs can also occur in mitotic phase G1 and could lead to different combination of duplication (head to head or tail to tail) (Fig. 5C). Chromosomic recombination via CRISMERE can also occur in a *cis* configuration in G2 leading directly to monosomic and trisomic daughter cells after mitosis (Fig. 5D). The mechanism would be similar to targeted asymmetric sister chromatid event of recombination TASCER^48^,^49^. Moreover, in rat and mouse founders, the variety of alleles is high and the size of the genomic region to rearrange does not seem to lead to consequences. Noteworthy, the orientation of the sgRNAs does not seem to favour one or the other rearrangement (data not shown).

CRISMERE is a powerful tool to generate duplication, deletion and inversion of different sizes, in different species and different genes or locus. All of the structural variants described in these paper will be valuable tools for the study of gene dosage in the context of Down syndrome. We anticipate that the only limit for CRISMERE will be the nature of the genomic region, i.e. the viability of its deletion, inversion, or duplication. Owning to the high efficiency of generating blunt end by Cas9, we showed that inversion, deletion and duplication of DNA fragments with defined length ranging in size from tens to thousands of kilobases can be easily achieved. Applying this technology to the editing of small DNA fragments such as millions of regulatory DNA elements^50^ is possible now. Also, generating mutations involving large DNA fragments of several hundred kilobases can be used to study complex gene clusters, the organization of chromosome in its genomic milieu, and copy number variants common in mammalian genomes^47^.

## Materials and Methods

All experiments were carried out in accordance with” relevant guidelines and regulations. Animal experiments were approved by the Com’Eth N°17 and accredited by the French Ministry for Superior Education and Research (“MESR”, MESR 00130 & MESR 01639), and were supervised by M.C.B. and YH who are qualified in compliance with the European Community guidelines for laboratory animal care and use (2010/63/UE) in our animal facility (Agreement C67-218-40).

### *In vitro* transcription of Cas9 mRNA and sgRNAs

We used the CRISPOR software (http://tefor.net/crispor/crispor.cgi), developed by the French TEFOR infrastructure, to select sgRNAs. Each sgRNA name refers to the specificity score given by the software. The Cas9 vector (T7-Cas9 wt cloned in pUC57) with T7 promoter was first linearized with AccI for use as a template for *in vitro* transcription with T7 polymerase. Cas9 mRNA was transcribed using mMESSAGEmMACHINE T7 Ultra Kit according to the manufacturer’s manual (Life Technologies). sgRNAs templates with T7 promoter were obtained by PCR amplification with primers (Supplementary Table S1). The MEGAshortscript Kit (Life Technologies) was used to transcribe sgRNAs from the PCR product template. Cas9 mRNA and sgRNAs were purified with the MEGAclear Kit (Life Technologies) and eluted in TE buffer (10 mM Tris-HCl, 0.1 mM EDTA, InVitrogen) for microinjections.

### *In vitro* cleavage assay to validate sgRNA’s functionality

Before injecting sgRNAs in eggs, all the guides were tested *in vitro* on the targeted DNA PCR product in order to validate their efficiency. PCR surrounding the targets were performed as described in *Mouse and rat genotyping for detecting DNA fragment inversions, duplications, and deletions* section below. Approximately 200 ng of target PCR product were mixed with 300 ng of Cas9 protein (PNA Bio Inc), 150 ng of sgRNA, 10X NEB buffer 3, 10X BSA and nuclease water qsp 10 μl. As control the Cas9 protein was omitted. The reactions were incubated at 37 °C for 1 h. One μl STOP solution (30% glycerol, 1.2% SDS, 250 mM EDTA, pH8.0) was added and the mix was incubated for 15 mins at 37 °C. The reaction was loaded on a 3% agarose gel. The correct sizes after Cas9 cleavage allow to validate the functionality of the tested guide. Only sgRNAs showing a cut (even partial) were injected in eggs.

### One-cell embryos injection

All mice were housed at 21 °C on a 14/12 h light-dark cycle (5:00am–07:00 pm) in the SPF facilities. Sexually immature female C57BL/6 N mice (4–5 weeks olds) were superovulated by intraperitoneal injection of 5 IU eCG followed by 2.5 IU hCG at an interval of 48 h and mated overnight with C57BL/6NCrl male mice that were >10 weeks old. Zygotes were collected after 20 h of hCG injection by oviductal flashing, and pronuclei-formed zygotes were put into the M2 medium (Sigma M-7167). Microinjection was performed using a microinjector (Eppendorf Femtojet 4i) equipped microscope. RNA solution was injected into the cytoplasm and the pronucleus of each zygote using continuous pneumatic pressure. After injection, embryos were *in vitro* cultured in the M16 medium (Sigma M-7292) at 37 °C in a 5% CO_2_ incubator. The survivors of the injected embryos were implanted into the oviducts of pseudo-pregnant CD1 mice.

All rats were housed at 21 °C on a 14/12 h light-dark cycle (5:00am–07:00 pm) in the SPF facilities. Sexually immature female Sprague Dawley rat (4–5 weeks olds) were superovulated by intraperitoneal injection of 20 IU eCG followed by 30 IU hCG at an interval of 48 h and mated overnight with Sprague Dawley male mice that were >10 weeks old. Zygotes were collected after 20 h of hCG injection by oviductal flashing, and pronuclei-formed zygotes were put into the M2 medium. Microinjection was performed using a microinjector equipped microscope. RNA solution was injected into the cytoplasm and the pronucleus of each zygote using continuous pneumatic pressure. After injection, embryos were *in vitro* cultured in the M16 medium at 37 °C in a 5% CO_2_ incubator. The survivors of the injected embryos were implanted into the oviducts of pseudo-pregnant CD1 mice.

### Mouse and rat genotyping for detecting DNA fragment inversions, duplications, and deletions

PCR was used to identify inversions, duplications, and deletions in mice with appropriate primer pairs (Supplementary Table S1). Those PCRs are performed with Phusion Taq HS (Fermentas) in a final volume of 20 μl. The PCR conditions are: predenaturing at 94 °C for 30 sec; followed by 35 cycles of 94 °C denaturation for 8 sec, 60 °C annealing for 10 sec, and 72 °C extension for 1 min; followed by a final extension at 72 °C for 5 min. Ten μl of these PCR products were denaturated, re-annealed and digested with T7 endonuclease in order to detect small deletions. Two size markers were used (GeneRuler 50 bp DNA ladder SM0372 and Generuler DNA ladder Mix SM0333).

### Droplet digital PCR validation and Copy number Variation

To confirm the deletion or the duplication in mice or rats, several ddPCR were performed using the QX200 droplet reader (Bio-Rad). Each ddPCR reaction contained duplex TaqMan assay reagents for the target and reference genes. The ddPCR reaction mixture consisted of 10 μl of a 2 × ddPCR Mastermix (Bio-Rad), 1 μl of target 20x primer/probe mix (IDT), 1 μl of reference 20× primer/probe mix (IDT), and 2 μl of DNA in a final volume of 20 μl. All primers and probes sequences are listed in Supplementary Table 2. The ratio between primer and probe was kept at 3:1. Primers and probes were designed with Prime time assays from Integrated DNA technologies or with Universal ProbeLibrary website from Roche Diagnostics. Each assembled ddPCR reaction mixture was then loaded into the sample well of an eight-channel disposable droplet generator cartridge (Bio-Rad). A volume of 70 μL of droplet generation oil (Bio-Rad) was loaded into the oil well for each channel. The cartridge was placed into the droplet generator (Bio-Rad). The cartridge was removed from the droplet generator, where the droplets that collected in the droplet well were then manually transferred with a multichannel pipet to a 96-well PCR plate. The plate was heat-sealed with a foil seal and then placed on a conventional thermal cycler and amplified to the end-point as follow: 95 °C for 10 min, followed by 40 cycles of 95 °C for 20 s and 58.2 °C for 60 s, 1 cycle of 98 °C for 10 min and ending at 12 °C. After PCR, the 96-well PCR plate was loaded on the droplet reader (Bio-Rad), which automatically reads the droplets from each well of the plate (32 wells/h). Analysis of the ddPCR data and CNV were performed with QuantaSoft analysis software (Bio-Rad) that accompanied the droplet reader.

## Additional Information

**How to cite this article:** Birling, M.-C. *et al*. Efficient and rapid generation of large genomic variants in rats and mice using CRISMERE. *Sci. Rep.* **7**, 43331; doi: 10.1038/srep43331 (2017).

**Publisher's note:** Springer Nature remains neutral with regard to jurisdictional claims in published maps and institutional affiliations.

## Supplementary Material

Supplementary Information

## Figures and Tables

**Table 1:** Recapitulates the projects (gene name and size of the genomic region), microinjection conditions, the numbers of eggs used and the results obtained.

Gene or region of interest	*Cbs*	*Dyrk1a*	*Umodl1-Prmt2* (1^st^)	*Umodl1-Prmt2* (2^d^)	*Lipi-Zfp295*	*Hmgn1*	*Tiam1*	*Runx1-Cbr1*
Organism	Rat	rat	rat	rat	rat	mouse	mouse	mouse
Interval between the two pairs of sgRNAs	37.2 kb	121.7 kb	3 513 kb	3 513 kb	24 499 kb	16.8 kb	226 kb	1 100 kb
Cas9 mRNA ng/μl	25–50	25–100	10–50	10–25	25–50	25–50	25–200	10–25
sgRNA ng/μl	12–25	12–50	10–50	10–12	10–50	12–25	25–100	12
Eggs injected	212	452	609	149	246	173	441	418
Eggs reimplanted	154	255	365	99	140	128	350	280
F0 newborns	24	28	40	15	9	8	41	34
F0 carriers with deletions detected	12 (5*)(3^§^)	4	2	1dd	1**	4 (2^§^)	8 (3^§^)	1^†,§^
F0 carriers with inversion detected	7 (2^§^)	3 (1^§^)	0	0 (1^§^)	0	0	0	1
F0 carriers with duplication detected	1 (1^§^)	2 (2^§^)	0	0 (1^§^)	1**	0	1 (2^§^)	0^§^
*with 2 different deletions observed				dd only by ddPCR	**stillborn	^§^GLT		^†^dead

F0 breedings are still ongoing at the time we write this paper and more germ line transmissions (GLTs) are expected in the next months.

## References

[b1] Dierssen M., Herault Y. & Estivill X. Aneuploidy: from a physiological mechanism of variance to Down syndrome. Physiol Rev 89, 887–920 (2009). 19584316 10.1152/physrev.00032.2007

[b2] Choong X. Y., Tosh J. L., Pulford L. J. & Fisher E. M. Dissecting Alzheimer disease in Down syndrome using mouse models. Front Behav Neurosci 9, 268 (2015). 26528151 10.3389/fnbeh.2015.00268 PMC4602094

[b3] Lana-Elola E. . Genetic dissection of Down syndrome-associated congenital heart defects using a new mouse mapping panel. Elife 5 (2016). 10.7554/eLife.11614 PMC4764572 26765563

[b4] Brault V. . Opposite phenotypes of muscle strength and locomotor function in mouse models of partial trisomy and monosomy 21 for the proximal Hspa13-App region. PLoS Genet 11, e1005062 (2015). 25803843 10.1371/journal.pgen.1005062 PMC4372517

[b5] Jiang X. . Genetic dissection of the Down syndrome critical region. Hum Mol Genet (2015). 10.1093/hmg/ddv364 PMC4614710 26374847

[b6] Olson L. E., Richtsmeier J. T., Leszl J. & Reeves R. H. A chromosome 21 critical region does not cause specific Down syndrome phenotypes. Science 306, 687–690 (2004). 15499018 10.1126/science.1098992 PMC4019810

[b7] Brudno M. . Automated whole-genome multiple alignment of rat, mouse, and human. Genome Res 14, 685–692 (2004). 15060011 10.1101/gr.2067704 PMC383314

[b8] Zhao S. . Human, mouse, and rat genome large-scale rearrangements: stability versus speciation. Genome Res 14, 1851–1860 (2004). 15364903 10.1101/gr.2663304 PMC524408

[b9] Mullins L. J. & Mullins J. J. Insights from the rat genome sequence. Genome Biol 5, 221 (2004). 15128437 10.1186/gb-2004-5-5-221 PMC416459

[b10] Nigam R. . Rat Genome Database: a unique resource for rat, human, and mouse quantitative trait locus data. Physiol Genomics 45, 809–816 (2013). 23881287 10.1152/physiolgenomics.00065.2013 PMC3783816

[b11] Zhang L. . Human chromosome 21 orthologous region on mouse chromosome 17 is a major determinant of Down syndrome-related developmental cognitive deficits. Hum Mol Genet 23, 578–589 (2014). 24041763 10.1093/hmg/ddt446 PMC3888256

[b12] Brault V., Pereira P., Duchon A. & Herault Y. Modeling chromosomes in mouse to explore the function of genes, genomic disorders, and chromosomal organization. PLoS Genet 2, e86 (2006). 16839184 10.1371/journal.pgen.0020086 PMC1500809

[b13] Brault V., Besson V., Magnol L., Duchon A. & Herault Y. Cre/loxP-mediated chromosome engineering of the mouse genome. Handb Exp Pharmacol 178, 29–48 (2007). 10.1007/978-3-540-35109-2_2 17203650

[b14] Herault Y., Rassoulzadegan M., Cuzin F. & Duboule D. Engineering chromosomes in mice through targeted meiotic recombination (TAMERE). Nat Genet 20, 381–384 (1998). 9843213 10.1038/3861

[b15] Ramirez-Solis R., Liu P. & Bradley A. Chromosome engineering in mice. Nature 378, 720–724 (1995). 7501018 10.1038/378720a0

[b16] Zheng B., Mills A. A. & Bradley A. A system for rapid generation of coat color-tagged knockouts and defined chromosomal rearrangements in mice. Nucleic Acids Res 27, 2354–2360 (1999). 10325425 10.1093/nar/27.11.2354 PMC148802

[b17] Adams D. J. . Mutagenic insertion and chromosome engineering resource (MICER). Nat Genet 36, 867–871 (2004). 15235602 10.1038/ng1388

[b18] Ruf S. . Large-scale analysis of the regulatory architecture of the mouse genome with a transposon-associated sensor. Nature Genetics 43, 379–386 (2011). 21423180 10.1038/ng.790

[b19] Spitz F., Herkenne C., Morris M. A. & Duboule D. Inversion-induced disruption of the Hoxd cluster leads to the partition of regulatory landscapes. Nat Genet 37, 889–893 (2005). 15995706 10.1038/ng1597

[b20] Li J. . Efficient inversions and duplications of mammalian regulatory DNA elements and gene clusters by CRISPR/Cas9. J Mol Cell Biol 7, 284–298 (2015). 25757625 10.1093/jmcb/mjv016 PMC4524425

[b21] Wang L. . Large genomic fragment deletion and functional gene cassette knock-in via Cas9 protein mediated genome editing in one-cell rodent embryos. Scientific Reports 5, 17517 (2015). 26620761 10.1038/srep17517 PMC4664917

[b22] Zhang L. . Large genomic fragment deletions and insertions in mouse using CRISPR/Cas9. PLoS One 10, e0120396 (2015). 25803037 10.1371/journal.pone.0120396 PMC4372442

[b23] Kraft K. . Deletions, Inversions, Duplications: Engineering of Structural Variants using CRISPR/Cas in Mice. Cell Rep 10, 833–339 (2015). 10.1016/j.celrep.2015.01.016 25660031

[b24] Boroviak K., Doe B., Banerjee R., Yang F. & Bradley A. Chromosome engineering in zygotes with CRISPR/Cas9. Genesis 54, 78–85 (2016). 26742453 10.1002/dvg.22915 PMC4819711

[b25] Lopes Pereira P. . A new mouse model for the trisomy of the Abcg1-U2af1 region reveals the complexity of the combinatorial genetic code of down syndrome. Hum Mol Genet 18, 4756–4769 (2009). 19783846 10.1093/hmg/ddp438 PMC2778371

[b26] Yu T. . Effects of individual segmental trisomies of human chromosome 21 syntenic regions on hippocampal long-term potentiation and cognitive behaviors in mice. Brain Research 1366, 162–171 (2010). 20932954 10.1016/j.brainres.2010.09.107 PMC3027718

[b27] Watanabe M. . Mice deficient in cystathionine beta-synthase: animal models for mild and severe homocyst(e)inemia. Proc Natl Acad Sci USA 92, 1585–1589 (1995). 7878023 10.1073/pnas.92.5.1585 PMC42564

[b28] De la Torre R. . Epigallocatechin-3-gallate, a DYRK1A inhibitor, rescues cognitive deficits in Down syndrome mouse models and in humans. Mol Nutr Food Res 58, 278–288 (2014). 24039182 10.1002/mnfr.201300325

[b29] Luco S. M. . Case report of novel DYRK1A mutations in 2 individuals with syndromic intellectual disability and a review of the literature. BMC Med Genet 17, 15 (2016). 26922654 10.1186/s12881-016-0276-4 PMC4769499

[b30] Bronicki L. M. . Ten new cases further delineate the syndromic intellectual disability phenotype caused by mutations in DYRK1A. Eur J Hum Genet 23, 1482–1487 (2015). 25920557 10.1038/ejhg.2015.29 PMC4613470

[b31] van Bon B. W. . Disruptive de novo mutations of DYRK1A lead to a syndromic form of autism and ID. Mol Psychiatry 21, 26–32 (2016). 10.1038/mp.2015.5 PMC4547916 25707398

[b32] Møller R. S. . Truncation of the Down syndrome candidate gene DYRK1A in two unrelated patients with microcephaly. Am J Hum Genet 82, 1165–1170 (2008). 18405873 10.1016/j.ajhg.2008.03.001 PMC2427221

[b33] Guedj F. . DYRK1A: a master regulatory protein controlling brain growth. Neurobiol Dis 46, 190–203 (2012). 22293606 10.1016/j.nbd.2012.01.007

[b34] Altafaj X. . Neurodevelopmental delay, motor abnormalities and cognitive deficits in transgenic mice overexpressing Dyrk1A (minibrain), a murine model of Down’s syndrome. Hum Mol Genet 10, 1915–1923 (2001). 11555628 10.1093/hmg/10.18.1915

[b35] Fotaki V. . Dyrk1A haploinsufficiency affects viability and causes developmental delay and abnormal brain morphology in mice. Mol Cell Biol 22, 6636–6647 (2002). 12192061 10.1128/MCB.22.18.6636-6647.2002 PMC135639

[b36] Benavides-Piccione R. . Alterations in the phenotype of neocortical pyramidal cells in the Dyrk1A+/− mouse. Neurobiol Dis 20, 115–122 (2005). 16137572 10.1016/j.nbd.2005.02.004

[b37] Fotaki V., Martinez De Lagran M., Estivill X., Arbones M. & Dierssen M. Haploinsufficiency of Dyrk1A in mice leads to specific alterations in the development and regulation of motor activity. Behav Neurosci 118, 815–821 (2004). 15301607 10.1037/0735-7044.118.4.815

[b38] Arque G. . Impaired spatial learning strategies and novel object recognition in mice haploinsufficient for the dual specificity tyrosine-regulated kinase-1A (Dyrk1A). PLoS One 3, e2575 (2008). 18648535 10.1371/journal.pone.0002575 PMC2481280

[b39] Li Z. . Duplication of the entire 22.9 Mb human chromosome 21 syntenic region on mouse chromosome 16 causes cardiovascular and gastrointestinal abnormalities. Hum Mol Genet 16, 1359–1366 (2007). 17412756 10.1093/hmg/ddm086

[b40] Deng T. . HMGN1 modulates nucleosome occupancy and DNase I hypersensitivity at the CpG island promoters of embryonic stem cells. Mol Cell Biol 33, 3377–3389 (2013). 23775126 10.1128/MCB.00435-13 PMC3753902

[b41] Lane A. A. . Triplication of a 21q22 region contributes to B cell transformation through HMGN1 overexpression and loss of histone H3 Lys27 trimethylation. Nat Genet 46, 618–623 (2014). 24747640 10.1038/ng.2949 PMC4040006

[b42] Malliri A. . Mice deficient in the Rac activator Tiam1 are resistant to Ras-induced skin tumours. Nature 417, 867–871 (2002). 12075356 10.1038/nature00848

[b43] Growney J. D. . Loss of Runx1 perturbs adult hematopoiesis and is associated with a myeloproliferative phenotype. Blood 106, 494–504 (2005). 15784726 10.1182/blood-2004-08-3280 PMC1895175

[b44] Wu S., Ying G., Wu Q. & Capecchi M. R. Toward simpler and faster genome-wide mutagenesis in mice. Nat Genet 39, 922–930 (2007). 17572674 10.1038/ng2060

[b45] Lee H. J., Kweon J., Kim E., Kim S. & Kim J. S. Targeted chromosomal duplications and inversions in the human genome using zinc finger nucleases. Genome Res 22, 539–548 (2012). 22183967 10.1101/gr.129635.111 PMC3290789

[b46] Gupta A. . Targeted chromosomal deletions and inversions in zebrafish. Genome Res 23, 1008–1017 (2013). 23478401 10.1101/gr.154070.112 PMC3668355

[b47] Pan D. & Zhang L. Tandemly arrayed genes in vertebrate genomes. Comp Funct Genomics, 545269 (2008). 18815629 10.1155/2008/545269 PMC2547482

[b48] Duchon A., Besson V., Pereira P. L., Magnol L. & Herault Y. Inducing segmental aneuploid mosaicism in the mouse through targeted asymmetric sister chromatid event of recombination. Genetics 180, 51–59 (2008). 18757940 10.1534/genetics.108.092312 PMC2535701

[b49] Herault Y. . Controlled somatic and germline copy number variation in the mouse model. Curr Genomics 11, 470–480 (2010). 21358991 10.2174/138920210793176038 PMC3018727

[b50] Tai D. J. . Engineering microdeletions and microduplications by targeting segmental duplications with CRISPR. Nat Neurosci 17 517–522 (2016). 10.1038/nn.4235 PMC4903018 26829649

