# Supplementary material for: Efficient and rapid generation of large genomic variants in rats and mice using CRISMERE
Source: Sci Rep. 2017 Mar 7;7:43331. doi: 10.1038/srep43331 (PMC5339700; doi:10.1038/srep43331)
Supplement: Supplementary Information [file srep43331-s1.pdf]

Supplementary information

Efficient and rapid generation of large genomic variants in rat and mice using CRISMERE

Marie-Christine Birling[1], Laurence Schaeffer[1], Philippe André[1], Loic Lindner[1], Damien Maréchal[1], Abdel Ayadi[1], Tania Sorg[1], Guillaume Pavlovic[1], and Yann Hérault[1,2,3,4,5*]

[1]PHENOMIN, Institut Clinique de la Souris (ICS), CNRS, INSERM, University of Strasbourg, 1 rue Laurent Fries, F-67404 Illkirch-Graffenstaden, France

[2] Institut de Génétique et de Biologie Moléculaire et Cellulaire, Illkirch, France

[3] Centre National de la Recherche Scientifique, UMR7104, Illkirch, France

[4] Institut National de la Santé et de la Recherche Médicale, U964, Illkirch, France

[5] Université de Strasbourg, Illkirch, France

*Corresponding author: Yann Hérault

Keywords: Copy number variation, neurodevelopmental disorder, intellectual disability, mouse model, trisomy 21

Running title: Chromosomal Engineering in rodents

Confirmation of chromosomal rearrangement by ddPCR

All the structural variant were confirmed by Sanger sequencing of the PCR amplified junctions. Standard end point PCR allows to detect most of the rearrangement events even in a very mosaic background. Once the genetic modification goes through germ line, meiotic segregation occurs and only one version is transmitted to the next generation. So after breeding F0 founders (even mosaics) with WT animals, the copy number will be stable. The WT allele (counting for one copy) provides a good control for the other variant (deletion or duplication). Counting the number of copies present in the genome at the F1 is thus straightforward. One copy must be counted when a deletion is transmitted and 3 copies must be counted when a duplication is transmitted. Inversion events can only be observed by standard junction PCR but the maintenance of 2 copies of the alleles must also be proven.

Droplet digital PCR reactions were conducted on some of the F0 founders and F1 pups. ddPCR copy counting confirmed the rearrangements detected by PCR analysis. At least 2 probes located at different sites of the region of interest were used. Many founders were mosaics which implies that in most cases, ddPCR could not help to elucidate the precise nature of the genomic rearrangement. However, in some case, the picture was already clear on the F0 founders. When a clear deletion can be detected by ddPCR on a F0 founder, this must mean that few or no duplication events has occurred at the same time and it might not be worth expecting (and testing) the duplication in F1.

S1 Rat F0 founders Cbs duplication, inversion and deletion sequence obtained by CRISPR with a double pair of sgRNAs

WT sequence at both extremity of Cbs

3’ of Cbs
GCCGTACATCTTGCAGTGACAGTGAGGGTGGAACAAGCCGAGGCTACCCAGCTGTTCAGCCACATTCA
CACCTTCAAGGGTGTGGGCTTCCATTAGTTGGCATTTTTTAAGGAAAAGTTGTGAAGCCGATTTTTC
ATTGTAACTTTTCATACGCCAAACTGTTTTGACGTAACAGGAGAATGATTCATGGGTGCAGACCTGTA
ACCTTTGCTGGTCCCACACTGTAAAACCCCTTTCAAGGTGTATCATGGGCACATCCAGGTGGACAGGA
TATGGTCCAGGGGTTGCAGGTCCCCAGCTTGTGCTTAGAGCAGTGGACCAAGGGGCCTCTTTCCTTCA
GAAGTGTGTCTGAGGGCTGGGGGTGAAGCTCTCACTTGCCTGGCATATACAGAACCCTGGGTTCAGTG
GCCAGCACCCATAAATGCGGCATAACCCCATAGTCCTGGCAGTTGGCAGTTAGAGGGTCAGGAAAGCA
GAAGGCCACGCTCAAACCTGACTGCATCCTAAATCTGAGACAGGAGAGACCTTCTCAGACAGAAGGAC
ATCTGAGATTAGGACTCTGGGTGGCCAGTGGCAGGAAAGGAAGTATTTGACTTTCAGAAGGAAGAGGC
CTGCCGTTGTTGTCTGCTGTCTGAGGTGAGTAGATGAGACCTTGGGCCACCAATACCAGGCTCTCAAG
GGTTTCCTTGGTGAGAAGGGAGAATCTGGCATCTAGTCGTCATCGAGCGTTAAAGTGACGTCCGTGGC
TA--------------------------37.2 KB-----------------------------
ACCCAGAGTGGGTTCCAGTCCAGGGTGAAGTTGTCCAGATGCCACATGTGAACTCTCAGAGGTAGCTA
AGTGTCACTCAGGCCTGGCATTGAGGTGTGGTGTGGCCGAGGTATGTCAACCCCTCTCAGAACCTAGG
CAGCAACACCCTAGCTCCCAGCACTGGTGGTTTAGGGCAAGAGACACCACACAGAAAGACCTCAGACA
ATGGTGAGTGATGGGACATTTTCACTCGGATCATATCCTGGGCATGTAAACACACGCCAGTCATCCTG
TCCCTCAGGCCCTGGCCTGAGGCTGGGTGTTCAAAGGGTTGTATAGCAGGACAATGGTTCATTGGCTG
TTGACCAGCAGAGAAAGGCAGAGACCCCACAAGCCTCGAGGAGAAACTCCTGGTCACTAAGGGTACTA
GAGAAGCTCCTTACCCTCCCTCACCCCTCACCTGCTGGGATTTTATGCCCCAGATTTAAATTTTTGTT
TATACCAAGTGATATAAACCTACTTCAAGGACACAGGGCTAGCGCTTTTTCTAAAAGTTGTAGACAAA
GACTTTACAAGACACACCAATACTGTATCACTACAAATT 5’ of Cbs

Rat F0-264 and F1 head to tail duplication junction sequence

ACCCAGAGTGGGTTCCAGTCCAGCTGTTCAGCCACATTCACACCTTCAAGGGTGTGGGCTTCCATTAG
TTGGCATTTTTTAAGGAAAAGTTGTGAAGCCGATTTTTCATTGTAACTTTTCATACGCCAAACTGTT
TTGACGTAACAGGAGAATGATTCATGGGTGCAGACCTGTAACCTTT

Rat F0-264 5’ inversion junction sequence

GCCGTACATCTTGCAGTGACAGTGAGACTGGAACCCACTCTGGGTG

Rat F0-264 and F1 3’ inversion junction sequence (549 bp from sgRNA46 and 265 bp from sgRNA56)

TAGCCACGGACGTCACTGGTCACTAAGGGTACTAGAGAAGCTCCTTACCCTCCCTCACCCCTCACCTG
CTGGGATTTTATGCCCCAGATTTAAATTTTTGTTTATACCAAGTGATATAAACCTACTTCAAGGACAC
AGGGCTAGCGCTTTTTCTAAAAGTTGTAGACAAAGACTTTACAAGACACACCAATACTGTATCACTAC
AAATT

Rat F0-274 and F1 (deletion junction comprises a supplementary deletion of 165 bp after sgRNA56)

GCCGTACATCTTGCAGTGACAGTGAGGGTGGAACAAGCCGAGGCTACCCAGCTGTTCAGCCACATTCA
CACCTTCAAGGGTGTGGGCTTCCATTAGTTGGCATTTTTTAAGGAAAAGTTGTGAAGCCGATTTTTC
ATTGTAACTTTTCATACGCCAAACTGTTTTGACGTAACAGGAGACTGGCCTGAGGCTGGGTGTTCAAA
GGGTTGTATAGCAGGACAATGGTTCATTGGCTGTTGACCAGCAGAGAAA

Rat F0-268 and F1 (main deletion junction)

GCCGTACATCTTGCAGTGACAGTGAGGGTGGAAGTTGTCCAGATGCCACATGTGAACTCTCAGAGGTA
GCTAAGTGTCACTCAGGCCTGGCATTGAGGTGTGGTGTGGCCGAGGTATGTCAACCCCTCTCAGAACC
TAGGCAGCAACACCCTAGCTCCCAGCACTGGTGGTTTAGGGCAAGAGACACCACACAGAAAGACCTCA
GACAATGGTGAGTGATGGGACATTTTCACTCGGATCATATCCTGGGCATGTAAACACACGCCAGTCAT
CCTGTCCCTCAGGCCCTGGCCTGAGGCTGGGTGTTCAAAGGGTTGTATAGCAGGACAATGGTTCATTG
GCTGTTGACCAGCAGAGAAAGGCAGAGACCCCACAAGCCTCGAGGAGAAACTCCTGGTCACTAAGGGT

ACTAGAGAAGCTCCTTACCCTCCCTCACCCCTCACCTGCTGGGATTTTATGCCCCAGATTTAAATTTT
TGTTTATACCAAGTGATATAAACCTACTTCAAGGACACAGGGCTAGCGCTTTTTCTAAAAGTTGTAGA
CAAAGACTTTACAAGACACACCAATACTGTATCACTACAAATT

Rat F0-278 (main deletion junction sequence: 37.2 kb deleted plus 363 bp after sgRNA56)

GCCGTACATCTTGCAGTGACAGTGAGGGTGGAATATAAACCTACTTCAAGGACACAGGGCTAGCGCTT
TTTCTAAAAGTTGTAGACAAAGACTTTACAAGACACACCAATACTGTATCACTACAAATT

Rat F0-278 and F1 (junction showing a precise Cas9 cut) and F1 germ line transmission (2d deletion junction sequence, faint band)

GCCGTACATCTTGCAGTGACAGTGAGGGTGGAACAAGCCGAGGCTACCCAGCTGTTCAGCCACATTCA
CACCTTCAAGGGTGTGGGCTTCCATTAGTTGGCATTTTTTAAGGAAAAAGTTGTGAAGCCGATTTTTC
ATTGTAACTTTTCATACGCCAAACTGTTTTGACGTAACAGGAGAATGATTTGAAGTTGTCCAGATGCC
ACATGTGAACTCTCAGAGGTAGCTAAGTGTCACTCAGGCCTGGCATTGAGGTGTGGTGTGGCCGAGGT
ATGTCAACCCCTCTCAGAACCTAGGCAGCAACACCCTAGCTCCCAGCACTGGTGGTTTAGGGCAAGAG
ACACCACACAGAAAGACCTCAGACAATGGTGAGTGATGGGACATTTTCACTCGGATCATATCCTGGGC
ATGTAAACACACGCCAGTCATCCTGTCCCTCAGGCCCTGGCCTGAGGCTGGGTGTTCAAAGGGTTGTA
TAGCAGGACAATGGTTCATTGGCTGTTGACCAGCAGAGAAAGGCAGAGACCCCACAAGCCTCGAGGAG
AAACTCCTGGTCACTAAGGGTACTAGAGAAGCTCCTTACCCTCCCTCACCCCTCACCTGCTGGGATTT
TATGCCCCAGATTTAAATTTTTGTTTATACCAAGTGATATAAACCTACTTCAAGGACACAGGGCTAGC
GCTTTTTCTAAAAGTTGTAGACAAAGACTTTACAAGACACACCAATACTGTATCACTACAAATT

Supplementary data S1 (A) Duplication, inversion, and deletion of the *Cbs* gene in rat (37.2 kb) by CRISPR with two pairs of sgRNAs. Shown are sequences of the upstream and downstream duplication junctions as well as deletion junctions in various F0 founders. Nucleotides in purple and red indicate the sgRNA recognition sequences on both extremities. Only one F0 founder with a duplication was detected and sequenced. Different alleles with the deletion of the whole gene were characterized: F0-274 has one single deleted allele; F0-268 is an example of this as two distinct alleles were determined by Sanger sequencing; F0-278 had also two distinct deletion alleles.

S2 Rat F0 founders sequences: Dyrk1a duplications, inversion and deletion obtained by CRISPR/Cas9 with a double pair of sgRNAs (121 Kb)

GGTTTCAGAGGAATAATGGTTAAGGTTTATTTGGTTATGTGGATTTTCAGAGTACATGTCTTATAGAA
GAACCCTGCCCTTCGTTCGGAGCCTCTGCCCTGGCCTGATGGTGGTGTCTGTGGTACTGTTCAAACAG
TTAGACTTAAGACCTCTGTCAGCATCTACACCAAGTTCTTGTAAAAATGGCCACAGAAAGTTCCTATT
TAACCTCAGAGAGGCATAAGCTCAGAAATTCTAGAAGGGTTGCCCCTGTGTCTGTGGGACAAGGACAC
ATATTTTGTATGCCTTGTGGATGTTGAAGAAATTGTTGTCTTTGAAGGAATAGAACTGAAGTGATGAT
-----121 KB-------
TAATGCATCCAGTGTGAGCAGGGCCTGAGGTGAAGGGTGGGCTTGTGCAGGTGACTGCAGGGCCTAGC
TGCTGTCATGCAGTCCTGAGCCGTGGAAGGGGAGTTGACAGACCCTGGGAAGTGCTAGAGTGGGAAGG
AGAAAAGGAGCCTCTCAGTGCAGCCAGAACAGCATGAGGGACAGACAAATGGGATAAAGGAACTGGGC
CCGAGGCAGTAGGATTCTGGCTTTCCTTAAAAAGACGAATGCTAGAGAGCAGGAACTTGGAGGGATAA

Rat F0-384 and F1 (head to tail duplication junction sequence)

TAATGCATCCAGTGTGAGCAGGGCCTGAGGTGAAGGGTGGGCTTGTGCAGGTGACTGCAGGGCCTAGC
TGCTGTCATGCTCAGAAATTCTAGAAGGGTTGCCCCTGTGTCTGTGGGACAAGGACACATATTTTGTA
TGCCTTGTGGATGTTGAAGAAATTGTTGTCTTTGAAGGAATAGAACTGAAGTGATGAT

Rat F0-386 and F1 (head to tail duplication junction sequence)

TAATGCATCCAGTGTGAGCAGGGCCTGAGGTGAAGGGTGGGCTTGTGCAGGTGACTGCAGGGCCTAGC
TGCTGTCATGCAGTCCTGAGGTGGTGTCTGTGGTACTGTTCAAACAGTTAGACTTAAGACCTCTGTCA
GCATCTACACCAAGTTCTTGTAAAAATGGCCACAGAAAGTTCCTATTTAACCTCAGAGAGGCATAAGC
TCAGAAATTCTAGAAGGGTTGCCCCTGTGTCTGTGGGACAAGGACACATATTTTGTATGCCTTGTGGA
TGTTGAAGAAATTGTTGTCTTTGAAGGAATAGAACTGAAGTGATGAT

Rat F0-355 and F1 (5' junction inversion sequence)

GGTTTCAGAGGAATAATGGTTAAGGTTTATTTGGTTATGTGGATTTTCAGAGTACATGTCTTATAGAA
GAACCCTGCCCTTCGTTCGGAGCCTCTGCCCTGGACTGCATGACAGCAGCTAGGCCCTGCAGTCACCT
GCACAAGCCCACCCTTCACCTCAGGCCCTGCTCACACTGGATGCATTA

Rat F0-355 and F1 (3' junction inversion sequence)

ATCATCACTTCAGTTCTATTCCTTCAAAGACAACAATTTCTTCAACATCCACAAGGCATACAAAATAT
GTGTCCTTGTCCCACAGACACAGGGGCAACCCTTCTAGAATTTCTGAGCTTATGCCTCTCTGAGGTTA
AATAGGAACTTTCTGTGGCCATTTTTACAAGAACTTGGTGTAGATGCTGACAGAGGTCTTAAGTCTAA
CTGTTTGAACAGTACCACAGACACCACCATCAGGACAGACATCAAATGGGATAAAGGAACTGGGCCCG
AGGCAGTAGGATTCTGGCTTTCCTTAAAAAGACGAATGCTAGAGAGCAGGAACTTGGAGGGATAA

Rat F0-378 (deletion junction; 672 and 1147 bps deleted from 5' and 3' most external sgRNAs)

TTGTCTTTGCCTAGCCCTAGATTATGTGACTCCTGTCGTCGAGGTGCAGCATCATCTGCTTCTTGAGT
TCTGCCAAATCCCCAGAAAATATACCTTGTAACTGTTTTCACACTCGTGGATTAGTGCCAGGCCTTTT
AAACTACAGTTTTATATATTCCCTCATATAACCTTCCAGTAGGAAATGTTACCCATTTTACAGATGAG
TAGACTGAGCCTAGAGGTCTGGGGTTCACTTGACACATTGTTGTGCTGGGTATGAGTGGCAGCAGCCT
GAGATCATTGGTACAACCTCTCAGTGCTATATTTCCCCCAGCGTTCACAGCCCCCTGCCTATGTGCAC
ACCAGCCTCCCAGCATTCCTTGTGCCCGAGAGTTCTGACATAGGCTGGCATTGAGAGTGC

Supplementary data S2 (A) Duplication, inversion, and deletion of the *Dyrk1a* gene (122 kb) by CRISPR with two pairs of sgRNAs in rat. Shown are sequences of duplication and deletion junctions as well as upstream and downstream inversion junctions in various F0 founders. Nucleotides in purple and red indicate the sgRNA recognition sequences on both extremities. Different alleles with the duplication of the whole gene were characterized: F0-384 and F0-386; F0-355 is an example of inversed Dyrk1a allele. F0-378 is an example of deleted allele. In the case of this specific deletion, a

less precise cut occurred as 672bp 5’ of the external 5’ sgRNA and 1147 bp 3’ of the external 3’ sgRNA were also detected (see also **Supplementary figure 1)**

S3 Rat F0 founders sequences: region ranging from Umodl1 to Prmt2 deletions and inversions obtained by CRISPR/Cas9 with a double pair of sgRNAs (3.6 Mb)

A Rat WT genomic DNA sequences at both extremities (1st approach)

Near Umodl1

TCCCGGATTTTGGGACTCAGGACTAAACACGCAAACAAACAAAGCTAGCCTGTTCCACCTCTGCCCCA
TGCCCGACCCTCCTACCCTGCCACTCTTCTGAAACAGTCATGTCATAGTTTTCTCAGCACCGCTTTAA
ATACTTAGTGACCACGTGAGCGTGAACGGCAGCTGGACCATCTGGCGTAGTGCCGTTCCCTCTTCTCT
CTTTACTAGTTGTCTTTTTTTGTTTAGACTAACCCCACACGGTTTCCAGTCACCAGATTTCTGCCGCC
TAAAAACGTCCGGTCAAAACTTGACTGCTGCTGTTGTTGCTAAGAGATTTATCT------3.6 MB—
TGTGTGATGTTTTACATTATAAATATATGTTGTAACTTTCAGGTTGTAACTTTCTGCCTGTTTGTCTG
GACTCCTTTTCTGTAATTATCCTTGCCAAATGTCCCTTAGTTCCCTTCACATGTGCTCACTCAATCCA
GCAGCAATGAGCTTCCAAAATAACAGATCCTGCCCTAACTTATACCTTACGATGTATTGAGAGACATG
GCCACTCCAGAGAGATCTTCCAGTCAGGAGAAGAGTAAGGCTGACCAAAAGACAAAAGGTGGCCAGGA
GGGTTAAAAAGAGAAGCAGTAGACAGAGTTCAG Near Prmt2

B Deletion sequences

Rat F0-334 deletion sequence

TCCCGGATTTTGGGACTCAGGACTAAACACGCAAACAAACAAAGCTAGCCTGTTCCACCTCTGCCCCA
TGCCCGACCCTCCTACCCTGCCACTCTTCTGAAACAGTCATGTCATAGTTTTCTCAGCACCGCTTTAA
ATACTTAGTGACCACGTGAGCGTGAACGGCAGCTGGACCAAAGACAAAAGGTGGCCAGGAGGGTTAAA
AAGAGAAGCAGTAGACAGAGTTCAG

Rat F0-335 deletion sequence

TCCCGGATTTTGGGACTCAGGACTAAACACGCAAACAAACAAAGCTAGCCTGTTCCACCTCTGCCCCA
TGCCCGACCCTCCTACCCTGCCACTCTTCTGAAACAGTCATGTCATAGTTTTCTCAGCACCGCTTTAA
ATACTTAGTGACCACGTGAGCGTGAACGGCAGCTGGACCATCAGGAGAAGAGTAAGGCTGACCAAAAG
ACAAAAGGTGGCCAGGAGGGTTAAAAAGAGAAGCAGTAGACAGAGTTCAG

C Rat WT genomic sequences at both extremities (2d approach)

Near Umodl1

TTCATTGTAGCATTGGAAACGTCCCAGACAGCTTTGCACGCAAATATTCATCGAATTAATCGTTGGTC
TGGTCAGTTTCTGAAGCACCATAAATACCGGACCATCGCGGAGACTCCTCTCGGATATCCTGCTGTTT
GACAGACTCAGGATGATATAGCAGCTAAGCAGAGAGTCCGGGTGCTGGTTCTGTGTGAGCTCCTGGCT
GCTGCACACCTCCGTGCCCTTGATGTTTGCCACCCGGAAGCTCACCAGACTCCACTCTGGTGCTTGGA
GGGTATGGGCGGCTGCCCTGCCCTCTCCCAGAGCAATCAGCTCAGGCCTCTGCAACAGCGACTCCATG
CTGGGTGCTTGCTGAAGGGCCTAGGACCAGCCCTTTTCAGTTACCACATGTTTCCAAGGGAGCTCAAG
GCTGCTGCAACCACCATGTCCTCCGTGCTCGCGGATTGGCTCACCAGGCGACTGTTGAGCTTGTAGGC
TGTGGGCTGAATGGCTGCCCCGCTTGTTTTTCTCTGTCTCCAATAGGGCAAGCGGCACAAATCGAGGC
CATGGTGACTCCATTGCAGGAGCTGACTGGCGAGCCTACATGGTTGGGACTCGTGTTTGCCAAGCGGC
ACATCGCCCTGTCGCCAGTGCTAGCGGACCCGGGACTCCTCAGCAACGACTTGTGTGCTTGCAGTCTG
CTGGCTGAGACCTGTAGCAGAGGCTATAGATTGCTCATTCCCCACGTGGGACTCTGTTCTACCGTCTT
TCCGTCCGATTTGGTGAGTCCACATCTTGAAAGCATCTTCCCATAAGCCCCCCCCCCCATCTATTGC
CAGCCCTGCAGACGCCTTCTCTGTCTCTCAGACTCAGCGGAGCTCTGCGATGGGTGCTGGTGTCTGCC
GCCTATTAATTTTCTACATTTAGTTTATTTCCGACTATGAAGGTAGCTGTGTGTTT-----3.6 MB-
GATGGGGAGCTGGCAGGCTGTGTCTGCTTCGCTTTGCAGCAGCTGGGCTCCTTCCACTAGCCTCCTCA
CTGTTTGTCCTGCAGCTCTCAGAAACCCAGGACTCCCCTTCCCTTGTTCTACAACCTTTCCCATCTGC
CCCACCTCTTCCAGATTCCTCCTCCTCTCACCAACCTCAGGCTGGACCATGTCCGTCCCATCTACCTC
CACCCCCTGACCCCACCCCTATTTAACATTTCATCTTCTGGATCTATTACCCCTCATTTGTTCCCCTC
TATTGCTATCACTGTTCCTCCCGAGAGAGACCCAGTTTCCCTAACCTCCCTCACTTCCAGCGCTAACT
TTCGGTCTCATCGTGGCCCATACTCGACTCAGGTCTCGGACTTTGATTTTCTGTTGTCTGAGCCACAG
ACCCTTCCTAGGAACGACTTAGAGGAACAGTAGCGCCCCTTCCTGATCGACCCACGTTCCTTTCTCTA
CCTCAGATCTGTAGCCATAAGATTCAGACTCCACCCCCAGCCCCGCCCCACCTTTTCTGATCAGCCCC

TAGCCCTAACTGCCTTCCCGGAATCTGTCTTCTGCACCCCATCGCATGACCTGGGATGGTTGCCAGTA
ACTCCTTCTGACTTCTGACTCTGAGAGGATGAGGTTGGAGCACAAAGTGCTCCAATCATAGCTGAGGT
GGCTTTGCCACCGGGTTCCAAGATGAACTTGTTAGTGACTCCGTCAACTTCAATGATGGCACGATGAG
GCAAGTATCTTCTTCTGTTTATTTTTTTAATGTCTATGGGTGTTTTGCCTGCATGTATGTCTGTGCGT
GAGACATTTGTGCCCGATGCCTGTGGAAGACGGAAGAGGGTACAGATCCACTGGAACCTCGGGAATTC
ACCATCTTTGTGAGCAACCAGGTGGATGCTGGGAATTGA Near Prmt2

D Sequence at the deletion junction (F1-290 born after breeding of F0-802)

TTCATTGTAGCATTGGAAACGTCCCAGACAGCTTTGCACGCAAATATTCATCGAATTAATCGTTGGTC
TGGTCAGTTTCTGAAGCACCATAAATACCGGACCATCGCGGAGACTCCTCTCGGATATCCTGCTGTTT
GACAGACTCAGGATGATATAGCAGCTAAGCAGAGAGTCCGGGTGCTGGTTCTGTGTGAGCTCCTGGCT
GCTGCACACCTCCGTGCCCTTGATGTTTGCCACCCGGAAGCTCACCAGACTCCACTCTGGTGCTTGGA
GGGTATGGGCGGCTGCCCTGCCCTCTCCCAGAGCAATCAGCTCAGGCCTCTGCAACAGCGACTCCATG
CTGGGTGCTTGCTGAAGGGCCTAGGACCAGCCCTTTTCAGTTACCACATGTTTCCAAGGGAGCTCAAG
GCTGCTGCAACCACCATGTCCTCCGTGCTCGCGGATTGGCTCACCAGGCGACTGTTGAGCTTGTAGGC
TGTGGGCTGATGGCTGCCCCGCTTGTTTTTCTCTGTCTCCAATAGGGCAAGCGGCACAAATCGAGGCC
ATGGTGACTCCATTGCAGGAGCTGACTGGCGAGCCTACATGGTTGGGACTCGTGTTTGCCAAGCGGCA
CATCGCCCTGTCGCCAGTGCTAGCGGACCCGGG*GCTTATGGGAAGATGCTTTCAAGATGTGGACTCAC*
*CAAATCGGACGGAAAGACGGTAGAACAGAGTC****CCA****C*CTTTTCTGATCAGCCCCTAGCCCTAACTGCCT
TCCCGGAATCTGTCTTCTGCACCCCATCGCATGACCTGGGATGGTTGCCAGTAACTCCTTCTGACTTC
TGACTCTGAGAGGATGAGGTTGGAGCACAAAGTGCTCCAATCATAGCTGAGGTGGCTTTGCCACCGGG
TTCCAAGATGAACTTGTTAGTGACTCCGTCAACTTCAATGATGGCACGATGAGGCAAGTATCTTCTTC
TGTTTATTTTTTTAATGTCTATGGGTGTTTTGCCTGCATGTATGTCTGTGCGTGAGACATTTGTGCCC
GATGCCTGTGGAAGACGGAAGAGGGTACAGATCCACTGGAACCTCGGGAATTCACCATCTTTGTGAGC
AACCAGGTGGATGCTGGGAATTGA

E Sequence at the duplication junction (F1-289 born after breeding of F0-802)

GATGGGGAGCTGGCAGGCTGTGTCTGCTTCGCTTTGCAGCAGCTGGGCTCCTTCCACTAGCCTCCTCA
CTGTTTGTCCTGCAGCTCTCAGAAACCCAGGACTCCCCTTCCCTTGTTCTACAACCTTTCCCATCTGC
CCCACCTCTTCCAGATTCCTCCTCCTCTCACCAACCTCAGGCTGGACCATGTCCGTCCCATCTACCTC
CACCCCCTGACCCCACCCCTATTTAACATTTCATCTTCTGGATCTATTACCCCTCATTTGTTCCCCTC
TATTGCTATCACTGTTCCTCCCGAGAGAGACCCAGTTTCCCTAACCTCCCTCACTTCCAGCGCTAACT
TTCGGTCTCATCGTGGCCCATACTCGACTCAGGTCTCGGACTTTGATTTTCTGTTGTCTGAGCCACAG
ACCCTTCCTAGGAACGACTTAGAGGAACAGTAGCACTCCTCAGCAACGACTTGTGTGCTTGCAGTCTG
CTGGCTGAGACCTGTAGCAGAGGCTATAGATTGCTCATTCCCGGGTCCGCTAGCACTGGCGACAGGGC
GATGTGCCGCTTGGCAAACACGAGTCCCAACCATGTAGGCTCGCCAGTCAGCTCCTGCAATGGAGTCA
CCATGGCCTCGATTTGTGCCGCTTGCCCTATTGGAGACAGAGAAAAACAAGCGGGGCAGCCATTCAGC
CCACAGCCTACAAGCTCAACAGTCGCCTGGTGAGCCAATCCGCGAGCACGGAGGACATGGTGGTTGCA
GCAGCCTTGAGCTCCCTTGGAAACATGTGGTAACTGAAAAGGGCTGGTCCTAGGCCCTTCAGCAAGCA
CCCAGCATGGAGTCACTGTTGCAGAGGCCTGAGCTGATTGCTCTGGGAGAGGGCAGGGCAGCCGCCCA
TACCCTCCAAGCACCAGAGTGGAGTCTGGTGAGCTTCCGGGTGGCAAACATCAAGGGCACGGAGGTGT
GCAGCAGCCAGGAGCTCACACAGAACCAGCACCCGGACTCTCTGCTTAGCTGCTATATCATCCTGAGT
CTGTCAAACAGCAGGATATCCGAGAGGAGTCTCCGCGATGGTCCGGTATTTATGGTGCTTCAGAAACT
GACCAGACCAACGATTAATTCTGTTCTACCGTCTTTCCGTCCGATTTGGTGAGTCCACATCTTGAAAG
CATCTTCCCATAAGCCCCCCCCCCCATCTATTGCCAGCCCTGCAGACGCCTTCTCTGTCTCTCAGAC
TCAGCGGAGCTCTGCGATGGGTGCTGGTGTCTGCCGCCTATTAATTTTCTACATTTAGTTTATTTCCG
ACTATGAAGGTAGCTGTGTGTTTGCGTGTGGGCATATGCACGCGAAAGGCCATGACAGAGGCCAGAAA
TGCCTGTTGGATCTCTTGGAGTTGGACTTACAGACATTGCCAGAAACCTGATGCAGGTGCTGGAAACT
GAACCCAGGTCCTCTGAAAAAAACCAAAAAACACACATGCTCTCAATCACAGAGACACATCCCTGCTC
TGTGATTAACCACAGGCCGAGTTCAGCCTTGCTCTCCAGGCTGGG

Supplementary Data S3 (A) Sequences at both extremities of the 3.6 Mb region (1st approach). Nucleotides in purple and red indicate the sgRNA recognition sequences on both extremities. The nucleotides in red indicate the sgRNA recognition sequence. The nucleotides underlined and in bold at PAMs. **(B)** Deletion of a 3.6 Mb DNA region by CRISPR with two pairs of sgRNAs in rat. Shown are sequences of the upstream and downstream duplication junctions as well as deletion junctions in

two rat founders F0-334 and F0335. **(C)** Sequence at both extremities of the 3.6 Mb region (2d approach). Nucleotides in purple and red indicate the sgRNA recognition sequences on both extremities. The nucleotides in red indicate the sgRNA recognition sequence. The nucleotides underlined and in bold at PAMs. The sequence in light green is found inversed at the duplication junction. The sequence in italic is found inversed at the deletion **(D)** Deletion of a 3.6 Mb DNA region by CRISPR with two pairs of sgRNAs in rat. Shown is the sequence of the deletion junction in F1 pups. Note that the sequence in italic correspond to an inversed 71 nts fragment located near the first sgRNA pair **(E)** Duplication of a 3.6 Mb DNA region by CRISPR with two pairs of sgRNAs in rat. Shown is the sequence of the duplication junction in F1 pups. Note that the sequence in light green correspond to the insertion of an inversed 590 nts fragment located near the first sgRNA pair.

S4

A Duplication and deletion in stillborn rat found by CRISPR with a double pair of sgRNAs

WT

Near Lipi
TATTTGTTGAAGGTC**CCA**ACTGGTAGTTGTATTGAGAGGTTTTAGAAGTTTTGGAGTTTAATAAAGAT
AGGTACA**TGG**TGGTCAGTATTG--------------------24,4 Mb-----------------
CTTCAGACAAAGTCTTATAGTA**CCA**CAGCATT*G*TAACCCAGTTTCTGAGGGAAAACTCTAGCCACCGG
GAACAGCTGGGCCAGGA Near Zfp295

Rat F0-2 Duplication

CTTCAGACAAAGTCTTATAGTA**CCA**CAGTTTCTGAGGGTCAGTATTG

Rat F0-2 Deletion

TATTTGTTGAAGCTATAGTAACCCAGTTTCTGAGGGAAAACTCTAGCCACCGGGAACAGCTGGG

B Indels at both extremities of the 24.4 Mb region

Stillborn indel 51 nts near Lipi (5')

TATTTGTTGAAGGTC**CCA**ACT----del51----ACA**TGG**TGGTCAGTATTG

Stillborn indel 35nts + insert 10nts near Zfp295 (3')

CTTCAGACAAAGTCTTATAGTA**CCA**CAG-del35-TACTCTAGCCACCGGGAACAGCTGGGCCAGGA

Supplementary Data S4 (A) Deletion and duplication of the 24.4 Mb DNA region by CRISPR with two pairs of sgRNAs in rat. Shown are sequences at both extremities of the WT genomic region as well as duplication and deletion junction sequences found in a rat (F0-2) stillborn founder. The nucleotides in red and purple indicate the sgRNAs recognition sequences 5' and 3' respectively. Light grey sequence indicate small insertions. **(B)** Indels at both 5' and 3' extremities at the 24.4 Mb region in the same stillborn founder. Shown are sequences observed near Lipi (5' extremity) with a 51 bps break point precisely at the expected Cas9 cutting site of each sgRNAs of the pair and near Zfp295 (3' extremity) with a precise 35 bps deletion accompanied with a 10 bps insertion.

S5 Mouse F0 founders and F1 of the mouse Hmgn1 gene: deletion obtained by CRISPR with a double pair of sgRNAs

A Hmgn1 mouse WT genomic sequence

CTGTCTCCACTGGACCCTGAATGCTTTCAACTCAGTGTATGAACACAAACCCACCTGCCTCCCCTGAA
ATCCTTGTGTCTTCCACTGCTTCTCCTTCCATCTTGCTGAGCAAACCACCAGCCGTCCATCCCATTCC
TGCCCTGTTTGACAG**GGG**CTTCCTTGGGTTCAGATGTGCACTT**CCT**CGGAAAGCTTGTGGTCCAGGAG
TGAGCCTATTTAGTTTGTGTCCTGTTCTCCTTCAATGTTCAGACTGGATGTGGAACAGTGAGTCAGGA
CAATAGACTACTGAGGTTCTGGGAAGAGCAGTCCTATGTTCACATGTTGTAATCTGGGTATGAAAAAG
----------------16.6kb---
TTAATTGCTCTGTGTGGCTTTTCACTCTCTCCTCTACCTTTTCCTTATCTGACAGCAATGCGAACTAC
AAAATAAGCTCAGTAAACTGAATTGAATGGATCTTTATGACATAGTGGCCTCAGTAGCTACTGAGGTA
AG**CCC**TACTTGGGTGAAGGAGTCCTGCCATCCCATGGCTAGGTCTTAGTATCTTAGTCATCATCAAAA
ACAAGCAGCTTACAAAGAAAATACACCATGGCTAGAGTCTTTGATATACA**AGG**TGTGAGTAAAAGCCA
ACAGCTTTGAAAACAGTCACTACTCAGGCATCTTTCTATCTAAAAATGTTTAGAACATCAGTGCTCCT
GCAAATGGCCTGGGTTCAGTTCCCAGCGCCTGCTGAGGAAACCATCTGTAACCTCGGTTCCAAGGATC

B Sequence of two deleted allele (F0-3 and F0-8) both GLT: established lines

FO-3 and F1 (line established)

CTGTCTCCACTGGACCCTGAATGCTTTCAACTCAGTGTATGAACACAAACCCACCTGCCTCCCCTGAA
ATCCTTGTGTCTTCCACTGCTTCTCCTTCCATCTTGCTGAGCAAACCACCAGCCGTCCATCCCATTCC
TGCCCTGTTTGACAG**GGG**CTTCCTTGGGTTCAGATGTGCACTT**C**ACA**AGG**TGTGAGTAAAAGCCAACA
GCTTTGAAAACAGTCACTACTCAGGCATCTTTCTATCTAAAAATGTTTAGAACATCAGTGCTCCTGCA
AATGGCCTGGGTTCAGTTCCCAGCGCCTGCTGAGGAAACCATCTGTAACCTCGGTTCCAAGGATC

FO-8 and F1 (line established) (A in bold inserted)

CTGTCTCCACTGGACCCTGAATGCTTTCAACTCAGTGTATGAACACAAACCCACCTGCCTCCCCTGAA
ATCCTTGTGTCTTCCACTGCTTCTCCTTCCATCTTGCTGAGCAAACCACCAGCCGTCCATCCCATTCC
TGCCCTGTTTG**A**ACAG**GGG**CTTCCTTGGGTTCAGATGTGCACTT**C**A**AGG**TGTGAGTAAAAGCCAACA
GCTTTGAAAACAGTCACTACTCAGGCATCTTTCTATCTAAAAATGTTTAGAACATCAGTGCTCCTGCA
AATGGCCTGGGTTCAGTTCCCAGCGCCTGCTGAGGAAACCATCTGTAACCTCGGTTCCAAGGATC

Supplementary Data S5 (A) Sequence at both extremities of the 16.6 kb *Hmgn1* gene. The nucleotides in red indicate the sgRNA recognition sequence. The nucleotides in bold are PAMs. The nucleotides in blue indicate the inserted sequences **(B)** Sequence of two distinct founder F0 (F0-3 and F0-8 and their germ lines.

S6 Mouse F0 founders and F1 of the mouse Tiam1 gene: duplication and deletion obtained by CRISPR with a double pair of sgRNAs

A Mouse WT genomic sequence

GAATCCCATGCCCTTCTTTAGTAACCTCCCGAGTCAGTGCAACAACAATTTTTACCTAACAGCCAAGG
GAGCGCCCATGTCTCCTCCCCAGAATTGATGTTGAAGCACGTGTTTATTCTGTCTGGCACCTTGTACC
ACCCTCATGGCCTCTGCTAATGTTCATTTATTTTTTTCTCAAGTGGTCCCGATATTATGTTCTCCTGC
TGTGGGGGACCCTGGGCTGGGCCTCCTTTGTGCCTCTTTGGCATATCTGATGGTTTGCTGAGTTTGGG
TACCCTCTGCCTGAGGGCTCCTTTTGCTCAGTTTTGCATACTGACGCCTGAAAGTGACCATGCTCACC
TCTTTGCTCCTGCGGACCCCAGGA----------------------226 KB----------------
CCAAGAATCCTATAGACATTTTAAGGATAAAGTTTATATAAAGTTGAGACTCAGTAGTTGGACCAGAG
ACATCCTAGAATCCAATTCTGGCTCCAGCAGATGGTTCATAGTTCAGTCTTAAACTCAGACAGATAGC
AATGTGGTCATGACT**CCA**GGAATAGTATCATTTGGTTTGTGGACGGAGGAAAAACCCAGGTAGGAGCG
GGGTT**GGG**AACAAGTTTTGTTCACTTGAAACAAATCTTTGATGCCTTTTATTAAAAGCCATCACATAA
AAAGGTTTGTTTGTAGAAAACCAGGGTTCTGGGGTTTCAACGAACCTTTCAAGTCTCAGGCAATGTTT
GGCCACTGATGGGAGAAAGGTGAACCAGACCCCAAAGAGCAATGATCCTGGATCCAAAATAT

B Sequence of the duplicated allele (F0-14 and F1**)**

CCAAGAATCCTATAGACATTTTAAGGATAAAGTTTATATAAAGTTGAGACTCAGTAGTTGGACCAGAG
ACATCCTAGAATCCAATTCTGGCTCCAGCAGATGGTTCATAGTTCAGTCTTAAACTCAGACAGATAGC
AATGTGGTCATGACT**CCA**GGAATAGTATCATTTGGTTTGGCCTCTGCTAATGTTCATTTATTTTTTTC
TCAAGTGGTCCCGATATTATGTTCTCCTGCTGTGGGGGACCCTGGGCTGGGCCTCCTTTGTGCCTCTT
TGGCATATCTGATGGTTTGCTGAGTTTGGGTACCCTCTGCCTGAGGGCTCCTTTTGCTCAGTTTTGCA
TACTGACGCCTGAAAGTGACCATGCTCACCTCTTTGCTCCTGCGGACCCCAGGA

C Sequence of the three deleted alleles

DEL F0-41 (226 kb) GLT: established line

GAATCCCATGCCCTTCTTTAGTAACCTCCCGAGTCAGTGCAACAACAATTTTTACCTAACAGCCAAGG
GAGCGCCCATGTCTCCTCCCCAGAACAAGTTTTGTTCACTTGAAACAAATCTTTGATGCCTTTTATTA
AAAGCCATCACATAAAAAGGTTTGTTTGTAGAAAACCAGGGTTCTGGGGTTTCAACGAACCTTTCAAG
TCTCAGGCAATGTTTGGCCACTGATGGGAGAAAGGTGAACCAGACCCCAAAGAGCAATGATCCTGGAT
CCAAAATAT

DEL F0-38 (226 kb + insertion of a repeated region 587 bp) GLT: established line

GAATCCCATGCCCTTCTTTAGTAACCTCCCGAGTCAGTGCAACAACAATTTTTACCTAACAGCCAAGG
GAGCGCCCATGTCTCCTCCCCAGAATTGATGTTGAAGCACGTGTTTATTCTGTCTGGCACCTTGTACC
AGAATAAACCAATATAAATATAAATACCAATTAAGGCCACGTAACCAGTTGCAGAAACGAGGATTATA
AAGTAATATGAATGCCCATTGTAAATTTACTAATGCGTTTGCGATTGTACGAGGGATAGTTATATCAT
GTTAGGCGTATTTACAGCCTTGTTATTGTTTCATGTGAACATGAGATATTATTTGTGTCAAGTTGACA
AGGGGTGGATTGTAGTGGCTATTCCTGGTTGTCAACTTGACAATATTTGGAATGAACTACAATTCGGA
ATTGGAAGGCTCACCAGTGACCCTTATCTGGAGGCTTGGAGATCCTTATCTGGATCTTGGTTTGAAGA
TCTTGAGCCATAGTGGCTATGGATTCCAGAAGATTGAATCTCCGAGTTAAGGAACACACCTTTAATCT
GGGCTATGCCTTTCATCTGGGATTAAAGGTGTGGTGGAACACACCTTTAATCTGGGCTACACCTTTTG
CTGGAGACAATATAAGGACATTGGAAGAAGGGAGTCTAGCTCTTGCTCTTGCTCTTGCTCCTTTGCTT
GCTTGCTGCGTGAGACTGAGTAACTGCTAGATCCTTGGACTTCTTATCATTTGGTTTGTGGACGGAGG
AAAAACCCAGGTAGGAGCGGGGTT**GGG**AACAAGTTTTGTTCACTTGAAACAAATCTTTGATGCCTTTT
ATTAAAAGCCATCACATAAAAAGGTTTGTTTGTAGAAAACCAGGGTTCTGGGGTTTCAACGAACCTTT
CAAGTCTCAGGCAATGTTTGGCCACTGATGGGAGAAAGGTGAACCAGACCCCAAAGAGCAATGATCCT
GGATCCAAAATATCTCTCTCCA

F0-40 (del226 kb + insertion 571 bp partial Syne2 mRNA from exon 57 to exon 71, both surrounding exons were not complete) GLT: established line

GAATCCCATGCCCTTCTTTAGTAACCTCCCGAGTCAGTGCAACAACAATTTTTACCTAACAGCCAAGG
GAGCGCCCATGTCTCCTCCCCAGAAGCTTGCAGTTCGTCAGCTGTAGGGCATCTAAAGTTATACACGC

TCACACTGGGTAGAGTACTTGTAGTTAGGGCCACCTGAGGCTCCACAAGCTGAGGTACGGAGGGATTT
GGTCTTGATGTTAGTTCATGGTGAAGATACTGCCACTTGTCACCGCTCTGGGCCTGAAGTGGCAAGGT
TGAATCTGATGCTGCATTCCCAGGACTAGGGACCTTGCTGGAAGCTGATTGCCGATTTGCATCTTCAT
TCTCTTGGTGACCCTGCAGCCATGTTTTCTTGGCATTCAGTTCTGTGAACTTGATTAAATCTTTCTGC
TCTGATGGTTTTAACTCCAGAATCTGTGGCTGGGGAGCATCCTTCTGTCTGCTGAACTGTGGCCTTTC
AGTTAGGACAGAGTCAAGCTCAGAAAGGCCAGCTGGCTGGTCGACGTCGTGGGGCTCTGACGGTTTCT
TAAGAGTGGAGGGATGCTGGTCCTCACTGAACTTCTCCTGAAGCACCATCTGGACCTTTTCCAAGTTG
CACTTCACAGTCTTGAGTTTCCAGGAAAGGGCCTCGGCCTCCTGCTGAGTGAT**GGG**AACAAGTTTTGT
TCACTTGAAACAAATCTTTGATGCCTTTTATTAAAAGCCATCACATAAAAAGGTTTGTTTGTAGAAAA
CCAGGGTTCTGGGGTTTCAACGAACCTTTCAAGTCTCAGGCAATGTTTGGCCACTGATGGGAGAAAGG
TGAACCAGACCCCAAAGAGCAATGATCCTGGATCCAAAATATCTCTCTCCA

Supplementary Figure S6 (A) Sequence at both extremities of the 226 kb *Tiam1* gene. The nucleotides in red indicate the sgRNA recognition sequence. The nucleotides in bold are PAMs. The nucleotides in blue indicate the inserted sequences **(B)** Sequence of the 3 deleted alleles. Deletion of a 226 kb DNA region by CRISPR with two pairs of sgRNAs in mouse. Shown are sequences of the upstream and downstream deletion junctions for three F0 founders. F0-41 sequence illustrates a whole deletion while F0-38 and F0-40 have exogenous DNA sequence inserted at the deletion site.

S7 Mice F0 founders sequences: region ranging from Runx1 to Cbr1 (16:16:92,601,466 to 93,610,505) 1Mb (R5897)

A Mouse WT genomic sequence at both extremities

TTAGTACATCAGCGACGTGCTCACGGTGCGCGAGTATTTCTAACACCGCAGGCAATGTACAAAGAA
CCTTCTGTGCTTCCCCGCAGCCTCAAAACAAACAAAAGTTTCCCACTTTCCGCTTTCGCAGCTTTGT
GAGAGGCACAAAGACATTTTGTAATTCACCCAAAGGTCCCGAGGGAGCCTG**CGG**TAGAT**CCA**CGGTTT
GATAGGGTTCAGTGTCTCTTGCTCTCTGATGGCCACAGATAGCAATCCTTTAGGGCAATGCAGAAGGA
ATCTGCAGGGTGTGGTCCAATGAATCTGTACGAAGCAAGACATTCAGACAGTGTTGAAACCGTTCCCC
TTTTCTCTTTCGAACGCATCTTAGGACTCTCGTTTCCCTTTGACAGCTTTATCCTGTGAATTGCATTT
------>1 MB---
CACTCTCCAGGGAGAGGCAGGCTGGCAATCAGCCCAAGAACTATGTCCTGCTCACGCACTGTAGTTTA
ACTTTTAGAAACACTGTTTGATTCTGCAGATCCCAGAGTGGAGGTCTCTT**CCC**CACGGTCAGTGCTTC
TGGGTGATATGAACCATCAGCTACTCAAGG**GGG**AGAGGGCTACAACTCTCCTCAGAAGGTGGAGAACC
TGCCCAGGCACAGATACAATGCTAAGGGTGGCCAGCAGAGGGCGCTGGGAAGGAAGGAAACACATTCA
CCAATAGGAGTCTAGAGCACTCTACCTGGCCCTGTCTGTGCACACATTGCTTCAAGGCCCTTTTTTTA
ATTTACTTACTAATTATTCCCTTGAATCTCTTTTTTTCCCATTTACTTCTTCCTTACTTTGTTCTCAG

B Sequence at the deletion junction (F0-32)

TTAGTACATCAGCGACGTGCTCACGGTGCGCGAGTATTTCTAACACCGCAGGCAATGTACAAAGAA
CCTTCTGTGCTTCCCCGCAGCCTCAAAACAAACAAAAGTTTCCCACTTTCCGCTTTCGCAGCTTTGT
GAGAGGCACAAAGACATTTTGTAATTCACCCAAAGGTCCCGAG**GGG**AGAGGGCTACAACTCTCCTCAG
AAGGTGGAGAACCTGCCCAGGCACAGATACAATGCTAAGGGTGGCCAGCAGAGGGCGCTGGGAAGGAA
GGAAACACATTCACCAATAGGAGTCTAGAGCACTCTACCTGGCCCTGTCTGTGCACACATTGCTTCAA
GGCCCTTTTTTTAATTTACTTACTAATTATTCCCTTGAATCTCTTTTTTTCCCATTTACTTCTTCCTT
ACTTTGTTCTCAG

C Sequence at the inversion junction (F0-7)

INV allele 5' sequence

TTAGTACATCAGCGACGTGCTCACGGTGCGCGAGTATTTCTAACACCGCAGGCAATGTACAAAGAA
CCTTCTGTGCTTCCCCGCAGCCTCAAAACAAACAAAAGTTTCCCACTTTCCGCTTTCGCAGCTTTGT
GAGAGGCACAAAGACATTTTGTAATTCACCCAAAGGTCCCGAGGGAGCCTG**CGG**TAGATCCACCCAAG
TTCAGTTAATAAGTTATATATGGAGTTCTCCCATGCATTGTGTGCCTACCAATCAAATCTTTACTGGA
TGAGACCTCCCAAAGATGTGTCCAGGAGTCCATGTACAATTTGAC

Supplementary Data S7 (A) Sequence at both extremities of the 1.1 Mb region spanning for *Runx1* to *Cbr1*. The nucleotides in red indicate the sgRNA recognition sequence. The nucleotides in bold are PAMs. **(B)** Deletion of a 1.1 Mb DNA region by CRISPR with two pairs of sgRNAs in rat. Shown is the sequence at deletion junction found in one F0 founder (F0-32) **(C)** Sequence of the 5' inversion junction confirming the inversion of the 1.1 Mb region.

Supplementary Table 1

	DNA fragment	sgRNAs	Direction	Sequence
Rat Cbs	3' of Cbs gene	sgRNA77	F	TAATACGACTCACTATAG**GAGTGAGGGTGGAACAAGCCG**GTTTTAGAGCTAGAAATAGCAAGTTAAAATAAGGCTAGTCCG
			R	AAAAGCACCGACTCGGTGCC
		sgRNA46	F	TAATACGACTCACTATAG**GGTAACAGGAGAATGATTCAT**GTTTTAGAGCTAGAAATAGCAAGTTAAAATAAGGCTAGTCCG
			R	AAAAGCACCGACTCGGTGCC
	5' of Cbs gene	sgRNA56	F	TAATACGACTCACTATAG**GTGCTGCCTAGGTTCTGAGAG**GTTTTAGAGCTAGAAATAGCAAGTTAAAATAAGGCTAGTCCG
			R	AAAAGCACCGACTCGGTGCC
		sgRNA51	F	TAATACGACTCACTATAG**GGCATCTGGACAACTTCACCC**GTTTTAGAGCTAGAAATAGCAAGTTAAAATAAGGCTAGTCCG
			R	AAAAGCACCGACTCGGTGCC
	Screening oligos	F1	F	GTTGACCCACCCATCTGGTGGGCTC
		R1	R	CTTGTGTCTGTCTAAATAGCTAGGT
		F2	F	AGAAACATGCCTCGTGGCTGATTGA
		R2	R	GCCACTGGCCACCCAGAGTCCTAAT
		F3	F	AACCTTTCTGGATCCTGTCATAAAG
		R3	R	AATTTGTAGTGATACAGTATTGGTG
		R4	R	TTTCTCTGCTGGTCAACAGCCAATG
Rat Dyrk1a	5' Dyrk1a	sgRNA50	F	TAATACGACTCACTATAGG**GTTCCTATTTAACCTCAGAG**GTTTTAGAGCTAGAAATAGCAAGTTAAAATAAGGCTAGTCCG
			R	AAAAGCACCGACTCGGTGCC
		sgRNA74	F	TAATACGACTCACTATAGG**CAGACACCACCATCAGGCCA**GTTTTAGAGCTAGAAATAGCAAGTTAAAATAAGGCTAGTCCG
			R	AAAAGCACCGACTCGGTGCC
	3' Dyrk1a	sgRNA82	F	TAATACGACTCACTATAGG**GTGCAGCCAGAACAGCATGA**GTTTTAGAGCTAGAAATAGCAAGTTAAAATAAGGCTAGTCCG
			R	AAAAGCACCGACTCGGTGCC
		sgRNA83	F	TAATACGACTCACTATAGG**TGTCATGCAGTCCTGAGCCG**GTTTTAGAGCTAGAAATAGCAAGTTAAAATAAGGCTAGTCCG
			R	AAAAGCACCGACTCGGTGCC
		F1	F	GAGACTGTTTGGGTACAGGTTTTGG

	Screening oligos	F2	F	GTACAAAATAGTTCTTGGTCATCCA
		R3	R	CTACGTTCGGGCCTTTGCCCTATCT
		F4	F	TGAGGGTTTACACTGCTAACAGAAC
		R4	R	CAATTAAGTTCAGTTCCCTTCTCTT
		F5	F	ATAACTATTAGGTACTGATGTTAAT
		R5	R	AGAAAACTCAGTGACAGAACACAAC
		F7	F	GGCAAAATTCGTGAGTCAGTGACTC
		R10	R	CTGCATCAATGCAGGTCTAGTGATG
Region between Umodl1 and Prmt2	5' Umodl1	sgRNA91	F	TAATACGACTCACTATAGG**AGCTGCCGTTCACGCTCACG**GTTTTAGAGCTAGAAATAGCAAGTTAAAATAAGGCTAGTCCG
			R	AAAAGCACCGACTCGGTGCC
		sgRNA95	F	TAATACGACTCACTATAGG**GGGAACGGCACTACGCCAGA**GTTTTAGAGCTAGAAATAGCAAGTTAAAATAAGGCTAGTCCG
			R	AAAAGCACCGACTCGGTGCC
		sgRNA68	F	TAATACGACTCACTATAGG**AGCTGCCGTTCACGCTCACG**GTTTTAGAGCTAGAAATAGCAAGTTAAAATAAGGCTAGTCCG
			R	AAAAGCACCGACTCGGTGCC
		sgRNA86	F	TAATACGACTCACTATAGG**TAGATTGCTCATTCCCCAC**GTTTTAGAGCTAGAAATAGCAAGTTAAAATAAGGCTAGTCCG
			R	AAAAGCACCGACTCGGTGCC
	3' Prmt2	sgRNA61	F	TAATACGACTCACTATAGG**TCCAGAGAGATCTTCCAGTC**GTTTTAGAGCTAGAAATAGCAAGTTAAAATAAGGCTAGTCCG
			R	AAAAGCACCGACTCGGTGCC
		sgRNA86	F	TAATACGACTCACTATAGG**CGTAAGGTATAAGTTAGGGC**GTTTTAGAGCTAGAAATAGCAAGTTAAAATAAGGCTAGTCCG
			R	AAAAGCACCGACTCGGTGCC
		sgRNA65		TAATACGACTCACTATAGG**AACGTGGGTCGATCAGGAAG**GTTTTAGAGCTAGAAATAGCAAG
			R	AAAAGCACCGACTCGGTGCC
		sgRNA61		TAATACGACTCACTATAGG**TGATCAGAAAAGGTGGGGCG**GTTTTAGAGCTAGAAATAGCAAG
			R	AAAAGCACCGACTCGGTGCC
	Screening oligos	F1	F	ATTGTACAAATTTATGAGGGTACAC
		R1	R	GGTATAGCCTGTCTTGGGAAATGTG

		F3	F	ACAGTCACAGGGAGGTCAAAGAGGGC
		R3	R	AGATAAATCTCTTAGCAACAACAGC
		F8	F	CGATGGGGAGCTGGCAGGCTGTGTC
		R9	R	GCAGACACCAGCACCCATCGCAGAG
		F13	F	CCTCCGTGCTCGCGGATTGGCTCAC
		R15	R	TCAGCTATGATTGGAGCACTTTGTG
Rat 24.4 Mb	5' Lipi	sgRNA80	F	GAAATTAATACGACTCACTATAGGTCTCAATACAACTACCAGTTGT TTTAGAGCTAGAAATAGCAAGTTAAAATAAGGCTAGTCCG
			R	AAAAAAAGCACCGACTCGGTGCCACTTTTTCAAGTTGATAACGGA CTAGCCTTATTTTAAC
		sgRNA62	F	GAAATTAATACGACTCACTATAGGGTTTAATAAAGATAGGTACAG TTTTAGAGCTAGAAATAGCAAGTTAAAATAAGGCTAGTCCG
			R	AAAAAAAGCACCGACTCGGTGCCACTTTTTCAAGTTGATAACGGA CTAGCCTTATTTTAAC
	3' Zfp295	sgRNA67	F	GAAATTAATACGACTCACTATAGG**GAAACTGGGTTACAATGCTG** GTTTTAGAGCTAGAAATAGCAAGTTAAAATAAGGCTAGTCCG
			R	AAAAAAAGCACCGACTCGGTGCCACTTTTTCAAGTTGATAACGGA CTAGCCTTATTTTAAC
		sgRNA73	F	GAAATTAATACGACTCACTATAGG**GAGGGAAAACTCTAGCCACC** GTTTTAGAGCTAGAAATAGCAAGTTAAAATAAGGCTAGTCCG
			R	AAAAAAAGCACCGACTCGGTGCCACTTTTTCAAGTTGATAACGGA CTAGCCTTATTTTAAC
	Screening oligos	PCR1	F	CCCAACCTCGGAAGGTGGACGCGAA
		PCR2	R	TGTTTCACCAACACGTGCCCAGGTA
		PCR5	F	GGTCACATTCAGCAAATTCTGTGTC
		PCR6	R	TTTGGGAACTGGACTCAGGAACAGG
Mouse Tiam1	5' of Tiam1	sgRNA69	F	GAAATTAATACGACTCACTATAGG**AAACCAAATGATACTATTCC**G TTTTAGAGCTAGAAATAGCAAGTTAAAATAAGGCTAGTCCG
			R	AAAAGCACCGACTCGGTGCC
		sgRNA70	F	GAAATTAATACGACTCACTATAGG**GGCACCTTGTACCACCCTCA**G TTTTAGAGCTAGAAATAGCAAGTTAAAATAAGGCTAGTCCG
			R	AAAAGCACCGACTCGGTGCC
	3' of Tiam1	sgRNA80	F	GAAATTAATACGACTCACTATAGG**ACGTGCTTCAACATCAATTC**G TTTTAGAGCTAGAAATAGCAAGTTAAAATAAGGCTAGTCCG
			R	AAAAGCACCGACTCGGTGCC

		SgRNA85	F	GAAATTAATACGACTCACTATAGGACCCAGGTAGGAGCGGGGTT GTTTTAGAGCTAGAAATAGCAAGTTAAAATAAGGCTAGTCCG
			R	AAAAGCACCGACTCGGTGCC
	Screening oligos	F1	F	TCTGCCAGCCGGCCTGCCTGTCTCA
		R1	R	CGCTTGTCTCCAGGCAACCACTGTC
		F2	F	AGAGAAGATGGGAAATGAGGATGCG
		R2	R	ACACAGGAAGTAGGTAGCAGGGGTC
		F3	F	CCAAGAATCCTATAGACATTTTAAG
		R3	R	AGGATCATTGCTCTTTGGGGTCTGG
		F4	F	TGGGGAAAGCCCAGCCAATGGTCTA
		R4	R	TTGTTGGCTTTCTCTGAGGCTCGTG
		R5	R	TTCAGAAACTGACATCACCATGAGC
		F6	F	GATACAAACCCCTGACTCCAGGCAG
		R6	R	TCCAGGTTCCTGCCCTGGATGATGG
Hmgn1	5' Hmgn1	sgRNA52	F	GAAATTAATACGACTCACTATAGGGCTAGAGTCTTTGATATACAG TTTTAGAGCTAGAAATAGCAAGTTAAAATAAGGCTAGTCCG
			R	AAAAGCACCGACTCGGTGCC
		sgRNA58	F	GAAATTAATACGACTCACTATAGGAGGACTCCTTCACCCAAGTAG TTTTAGAGCTAGAAATAGCAAGTTAAAATAAGGCTAGTCCG
			R	AAAAGCACCGACTCGGTGCC
	3' Hmgn1	sgRNA76	F	GAAATTAATACGACTCACTATAGGATTCCTGCCCTGTTTGACAGGT TTTAGAGCTAGAAATAGCAAGTTAAAATAAGGCTAGTCCG
			R	AAAAGCACCGACTCGGTGCC
		sgRNA81	F	GAAATTAATACGACTCACTATAGGCCTGGACCACAAGCTTTCCGG TTTTAGAGCTAGAAATAGCAAGTTAAAATAAGGCTAGTCCG
			R	AAAAGCACCGACTCGGTGCC
	Screening oligos	F1	F	GATGCTGCCCGTGACTCCATCTCAT
		R1	R	CTTGATGGCTTCATCGTCAGGCTGG
		F2	F	CCTTATCTGACAGCAATGCGAACTA
		R2	R	AGATAGAAAGATGCCTGAGTAGTGA
Region 16P11 BP2-BP3 (Lat - Atxn2l) 179kb	3' Lat	sgRNA45	F	TTAATACGACTCACTATAGGCATGGTTGTAGGTGATGTGGTTTTA GAGCTAGAAATAGC
				AAAAGCACCGACTCGGTGCC
		sgRNA50	F	TTAATACGACTCACTATAGGTGCAGGTGGGAAAACTCTGGTTTTA GAGCTAGAAATAGC

				AAAAGCACCGACTCGGTGCC
	5' Atxn2l	sgRNA71	F	TTAATACGACTCACTATAGG**TACCAGCTCAGAGGCTTGT**GTTTTAGAGCTAGAAATAGC
				AAAAGCACCGACTCGGTGCC
		sgRNA86	F	TTAATACGACTCACTATAGG**CTGTAGGCTAAACAGCAGA**GTTTTAGAGCTAGAAATAGC
				AAAAGCACCGACTCGGTGCC
	Screening oligos	PCR1	F	AGGTTCCTTGCAAGAAGGCAGACGT
		PCR2	R	GGGTGGCTGTGGAAGGCTCTGAGTT
		PCR11	F	GGTTAGAGTGTGCACTAGAGAGAAG
		PCR12	R	GTGCTCAGGTCTCTTCTGCATGTTG

Supplementary Table 2

	Forward primers	Reverse primers	Probes
**Tiam1**	AGAGACTTGGGTGAGAGACTTA	GTGCTTGCTACCCTCAGTAAA	/56-FAM/TTGTGCTTC/Zen/TGATGTGGAGTGGCT/3IABkFQ/[1]
	ATGGGAAACTGAAGCAGAGG	CCTCTCTAAGCACCAGACTTTC	/56-FAM/TTTCTGGTC/Zen/TCCCTGCAACACGTC/3IABkFQ/[1]
	GTAGACAGCAGGGTTGCATTA	AGGAGACCAGGAGAGTCTTATC	/5HEX/TTCCTCCCA/Zen/CACACATCTTGAC GG/3IABkFQ/[1]
**Dyrk1a**	CGCAGCGTAAGTATAACATTCAA	TGTGTCTAATACATGACACAGTCAGAA	Universal Probe Library #4[2]
	TGTCACAATGCTACCCCTACA	GAGCTTCAGTGCCTGACCA	Universal Probe Library #82[2]
	ACTGTCGCTCTCTGCAATTAG	GAGAGATGGCTCAGTGGTTAAG	/5HEX/TGTGGTCCT/Zen/GGGAATTGAACTCAGG/3IABkFQ/[1]
**Abcg1**	GGCATGTGTGACTCTGACTATAA	GGCCTCTCCAAACCTTCTTTA	/56-FAM/AGACGTGAAC/ZEN/CCGTTCCTCTGG/3IABkFQ/[1]
**Pde9a**	CAAGGCCATCTACCTAGACATC	GGACACCTGCGCCTATTA	/56-FAM/ATGGACGCAT/ZEN/CCAGAAGGTACCC/3IABkFQ/[1]
**Cbs**	TAGACTCAGACGCGGGAAA	CAATCACCTGAGCCTGTTGT	/56-FAM/TCGGACAGGA/ZEN/TCACAGCTCAGATAGG/3IABkFQ/[1]
**Sik1**	AGGTCCTCAGCATGTCTTTG	GCCCGTTGGAAGTCAGATAA	/56-FAM/AAGTCACACC/ZEN/AGAAGACGCTGAGC/3IABkFQ/[1]
**Trpm2**	AGTCAATAGGTGCGTGCTAAG	TTCTTCTCCAGCTTGTTCTCTG	/56-FAM/CCTGGGTGAG/ZEN/TTAACCCTTTCCGG/3IABkFQ/[1]
**Itgb2**	CCAGATCATGAGGTCACATACC	GCTGCTGGATAACCTACACTT	/56-FAM/TGTAAGCGT/ZEN/TTCCGTCCTTCTGCT/3IABkFQ/[1]
**Slc19a1**	TCACCTACTATGTCCATGTCCT	CTCCAGTGCATACTCAGAAGTG	/56-FAM/AGCAGACTCA/ZEN/ACTACAACGGTGCC/3IABkFQ/[1]
**Lss**	GTCGGCAGAGATGGACTTATT	GCTTCCAGACTTGGGATGAA	/56-FAM/CCCACTCTTT/ZEN/GGGACTGGACACAG/3IABkFQ/[1]
**S100b**	CATCAACAACGAGCTCTCTCA	CAGAGGCAGAAACGGAAGTTA	/56-FAM/CTGAGCTGCT/ZEN/CCAGAAATGGTCCT/3IABkFQ/[1]
**Glo1**	AAGGACAAGACGGAGAGGA	GGACTGGTGAGCAGAGAATAAA	/5HEX/TGTTTCCATC/ZEN/TCTTTCCGCAGCCT/3IABkFQ/[1]

[1] : ZEN^{TM} probes are double-quenched probes labelled with a specific fluorochrome. They can be ordered at http://eu.idtdna.com/

[2] : UPL are short hydrolysis probes (labelled at 5' end with fluorescein (FAM) and at the 3' end with a dark quencher dye) with some nucleotides modified with Locked Nucleic Acids (LNA) technique. UPL probes can be ordered at https://lifescience.roche.com/

Supplementary figure 1 (A) The loss of one complete copy was confirmed by ddPCR copy counting for F0-356, -368, -378 and F0-385. F0 founders with monosomic Dyrk1a are also smaller and lighter. The F0 founder's weights were taken at 12 weeks of age **(B)** Scheme of Dyrk1a locus with position of the sgRNAs, ddPCR primer and deletion junction PCR primers. The 5' and 3' details (repeated regions in yellow) show that deletion occurred farther than expected (respectively 627 and 1147 bp from the most external sgRNA). Oligonucleotides F5 and R4 could not detect the deletion as their sequences are located in the deleted region. The sequences corresponding to F7 and R10 are were not deleted so a PCR fragment diagnosed the deletion junction **(C)** to **(E)** Germ line transmission is confirmed for F0-378. **(C)** One copy of Dyrk1a was detected by ddPCR on F1 (-624, 625 and 626). **(D)** Deletion of a copy was also confirmed by PCR and junction sequencing. **(E)** At 4 weeks of age, monosomic rats show a 40 % weight reduction compared to *Wt* littermates.

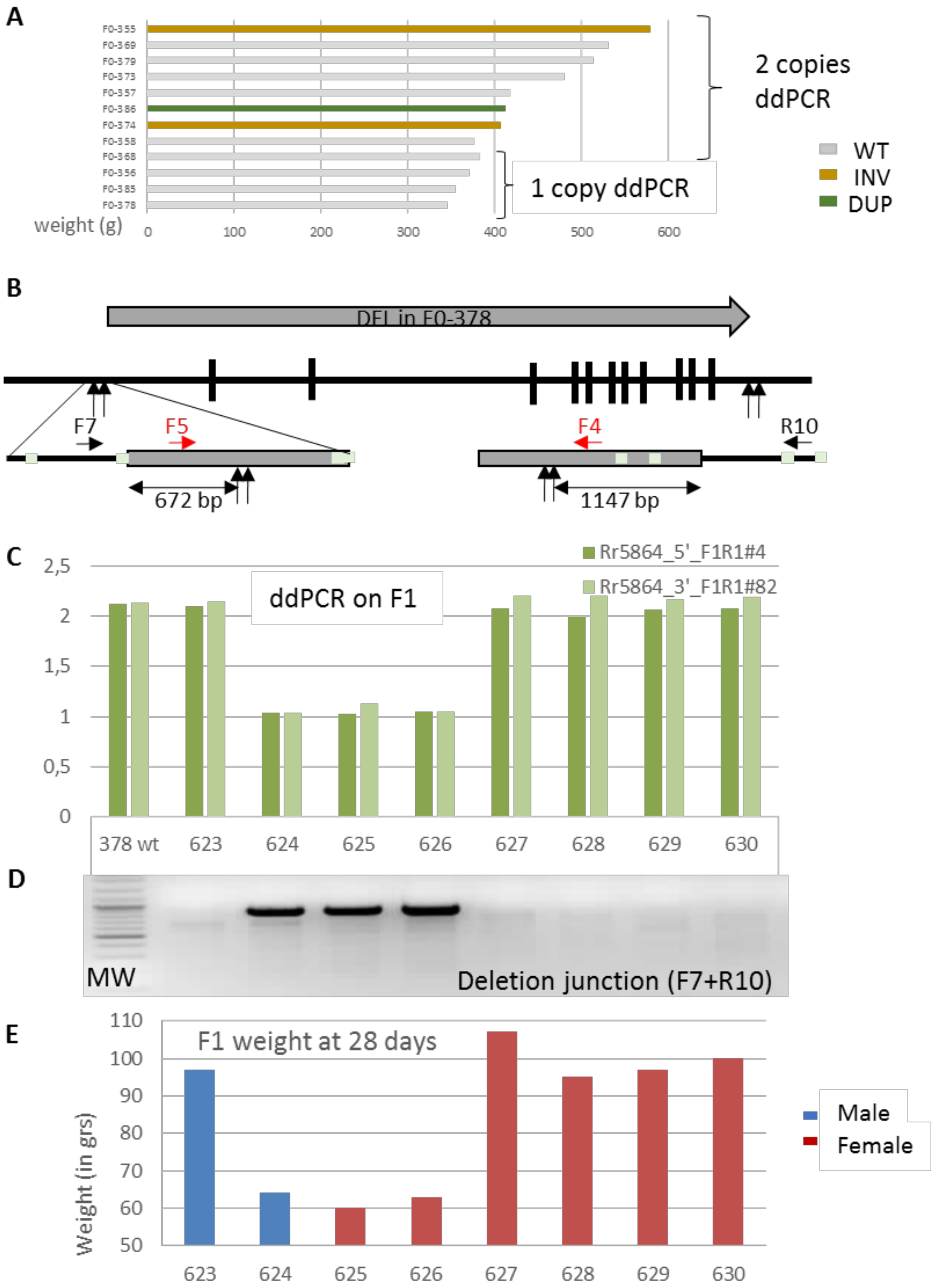
A
F0-355
F0-369
F0-379
F0-373
F0-357
F0-386
F0-374
F0-358
F0-368
F0-356
F0-385
F0-378
weight (g)
0
100
200
300
400
500
600
2 copies ddPCR
1 copy ddPCR
WT
INV
DUP
B
DEL in F0-378
F7
F5
F4
R10
672 bp
1147 bp
C
ddPCR on F1
Rr5864_5'_F1R1#4
Rr5864_3'_F1R1#82
2,5
2
1,5
1
0,5
0
378 wt
623
624
625
626
627
628
629
630
D
MW
Deletion junction (F7+R10)
E
F1 weight at 28 days
Weight (in grs)
110
100
90
80
70
60
50
623
624
625
626
627
628
629
630
Male
Female
